# Danger-Sensing/Patten Recognition Receptors and Neuroinflammation in Alzheimer’s Disease

**DOI:** 10.3390/ijms21239036

**Published:** 2020-11-27

**Authors:** Anna Chiarini, Ubaldo Armato, Peng Hu, Ilaria Dal Prà

**Affiliations:** 1 Section of Human Histology & Embryology, Department of Surgery, Dentistry, Pediatrics, and Gynecology, University of Verona, Verona, 37134 Venetia, Italy; peng.hu@univr.it; 2 Department of Burns and Plastic Surgery, University of Shenzhen, Shenzhen 518000, China

**Keywords:** Alzheimer’s disease, neuroinflammation, pattern recognition receptors, danger-sensing receptors, inflammasomes, calcium signaling

## Abstract

Fibrillar aggregates and soluble oligomers of both Amyloid-β peptides (Aβs) and hyperphosphorylated Tau proteins (p-Tau-es), as well as a chronic neuroinflammation are the main drivers causing progressive neuronal losses and dementia in Alzheimer’s disease (AD). However, the underlying pathogenetic mechanisms are still much disputed. Several endogenous neurotoxic ligands, including Aβs, and/or p-Tau-es activate innate immunity-related danger-sensing/pattern recognition receptors (PPRs) thereby advancing AD’s neuroinflammation and progression. The major PRR families involved include scavenger, Toll-like, NOD-like, AIM2-like, RIG-like, and CLEC-2 receptors, plus the calcium-sensing receptor (CaSR). This quite intricate picture stresses the need to identify the pathogenetically topmost Aβ-activated PRR, whose signaling would trigger AD’s three main drivers and their intra-brain spread. In theory, the candidate might belong to any PRR family. However, results of preclinical studies using in vitro nontumorigenic human cortical neurons and astrocytes and in vivo AD-model animals have started converging on the CaSR as the pathogenetically upmost PRR candidate. In fact, the CaSR binds both Ca^2+^ and Aβs and promotes the spread of both Ca^2+^ dyshomeostasis and AD’s three main drivers, causing a progressive neurons’ death. Since CaSR’s negative allosteric modulators block all these effects, CaSR’s candidacy for topmost pathogenetic PRR has assumed a growing therapeutic potential worth clinical testing.

## 1. Alzheimer’s Disease (AD)

Alzheimer’s disease (AD) is a devastating neurodegenerative illness that slowly yet diffusely kills neurons in cognitively critical cerebral cortical areas, eventually inducing the world’s most prevalent form of dementia. Medical advances have both increased the aging fraction of the human population and raised AD’s prevalence. Nowadays, AD, particularly in its sporadic (SAD) or late-onset (LOAD) form, affects about 35 million people, a figure likely to keep rising in the future. Hence, AD has become and will continue being a growingly serious familial, healthcare, and societal burden until the discovery of an effective therapy [1,2]. AD’s neuropathology leans on a triad of hallmarks: (i) extracellular congophilic plaques of insoluble fibrillar amyloid-β peptides (fAβs); (ii) intracellular insoluble aggregates (neurofibrillary tangles or NFTs) of hyperphosphorylated Tau proteins (p-Taues); and (iii) a diffuse chronic neuroinflammation [3,4]. But other nearly undetectable factors asymptomatically (for most of the time) yet relentlessly drive AD’s progression, such as soluble Aβ oligomers (sAβ-os) [5]; soluble p-Tau oligomers (p-Tau-os) [6]; reactive oxygen species (ROS) [7,8]; nitric oxide (NO) and its peroxynitrite derivative (ONOO^−^) [9]; vascular endothelial growth factor-A (VEGF-A) [10]; and a set of proinflammatory cytokines, chemokines, and other toxic agents [11,12,13]. The joint actions of such neurotoxins cause growing synaptic losses, neural circuits breakdowns, and human neurons and oligodendrocytes deaths, all happening within a chronically spreading neuroinflammation. The AD neuropathology’s clinical counterparts are steadily aggravating losses of memories and cognitive faculties that inexorably lead to patients’ dementia and ultimate demise [14,15,16,17,18]. Attempts are under course to identify according to scientific criteria specific markers that should determine the stages of the disease [2]. Hitherto, no therapeutic agents, including FDA-approved donepezil, a cholinesterase inhibitor, and memantine, an NMDA receptor antagonist, given singly or in combination, could alter AD’s inexorable progression [19].

Advances in science and medical technology have led to an increased debate on the pathophysiology of this type of dementia and to suggest several hypotheses about its pathogenesis (see Table 1).

So far, the detailed pathogenesis of SAD/LOAD, which develops into a clinical disease over the course of decades, is still debated due to (i) the complexity of human brains; (ii) the lack of specific diagnostic biomarkers useful for a for early diagnosis; and (iii) the interplay among several potential risk factors (Table 2).

The first research endeavors on AD focused on the pathogenetic roles played by Aβ peptides (Aβs) or p-Tau-es, the alternatively primary AD drivers [17,18]. Among a deluge of other hypotheses (Table 1), it was recently set forth that cells of all types in the senescing central nervous system (CNS) might convert the normal ageing process into a neurodegenerative illness [49]. Structural chromatin modifications, irreversible mitotic cell cycle arrest, downregulated expression of lamin B1 and neurotrophic factors, overexpression and overrelease of proinflammatory agents (e.g., IL-6, etc.) are held to characterize the senescent astrocytes (for further details and references see [50]). However, yet no agreed firm definition exists about cellular senescence and its triggering mechanisms under the diverse neuropathological conditions. Therefore, it still unclear whether cellular senescence is the cause or outcome of neurodegenerative processes [48]. At any rate, it is remarkable that, whatever the pathogenetic hypothesis be, AD always entails a chronic neuroinflammation. This fact has engendered the “Neuroimmunomodulation Hypothesis of AD”, implicating neuroinflammatory phenomena as primary causes of AD [35]. Consequently, the mechanisms underlying AD’s neuroinflammation have been attracting an increasing attention [3,4]. Clearly, the clarification of such neuroinflammatory mechanisms and the all-important approaches to counter or mitigate them might hopefully lead to novel and effective treatments of human AD [51].

## 2. Glia Roles in AD

According to the “Amyloid Hypothesis of AD”, the neuropathology starts within the layer II of the temporal lobe lateral entorhinal allocortex and thence via its neuritic projections spreads out towards cognitively crucial upper neocortical areas [52]. The slowly growing load of sAβ_42_-os and of insoluble Aβ_42_ fibrils drives the activation of astrocytes and microglia in the brain areas affected. Macroglia (astrocytes and oligodendrocytes) and microglia partake in the innate immune system of the CNS. Microglia are the resident cells carrying out the “immune surveillance”. In a “resting” or anti-inflammatory phenotype they keep brain homeostasis by secreting anti-inflammatory cytokines, such as TGF-β and IL-10, and neurotrophic factors such as BDNF and GDNF. Thus, homeostatic microglia promote differentiation and survival of neurons, favor learning-dependent synapse formation and plasticity, scavenge neuronal debris, and remove defective neurons by inducing their death [53,54]. Conversely, several stimuli, such as tissue damage, exogenous pathogens or endogenous protein aggregates turn on the microglia’s activated or proinflammatory phenotype, which gets rid of them through phagocytosis, and secretion of proinflammatory cytokines, such as TNF-α, IL-6, and IL-1β, and of cytotoxic factors such NO and ROS. The role microglia play in AD is quite complex and heavily debated, with conflicting reports regarding their harmful or shielding impact onto the disease; the most commonly held view being that microglia undergoes a potentially beneficial activation in early disease stages and a detrimental activation in later stages [55]. At the early stage, as shown in transgenic AD-model mice, activated microglia can in part exert beneficial effects on AD pathogenesis, clearing the Aβ-os through phagocytosis [56]. On the contrary, at a later stage the accrual of activated microglia mostly around Aβ plaques, as seen in tissue samples from AD human brains and AD-model animals, alongside with an increased production of proinflammatory factors, is ultimately noxious to surrounding neurons, thereby advancing disease progression [57].

Astrocytes are the most abundant CNS cell type, from 1.7-fold to 10-fold the neurons’ numbers [58] and are the key cell type helping keep CNS homeostasis. Some authors posit that the Aβ-elicited reactive astrogliosis is the leading driver of AD’s neuroinflammation [59]. Notably, the proinflammatory activity of the Aβ-driven astrocytes outlasts that of the less plentiful microglia [60,61]. It is important to realize that, once Aβ-activated, astrocytes and microglia reciprocally interact, which further increases the release of a complex set of proinflammatory cytokines, chemokines, and other neurotoxic factors from either cell type [62,63].

**Figure 1:**
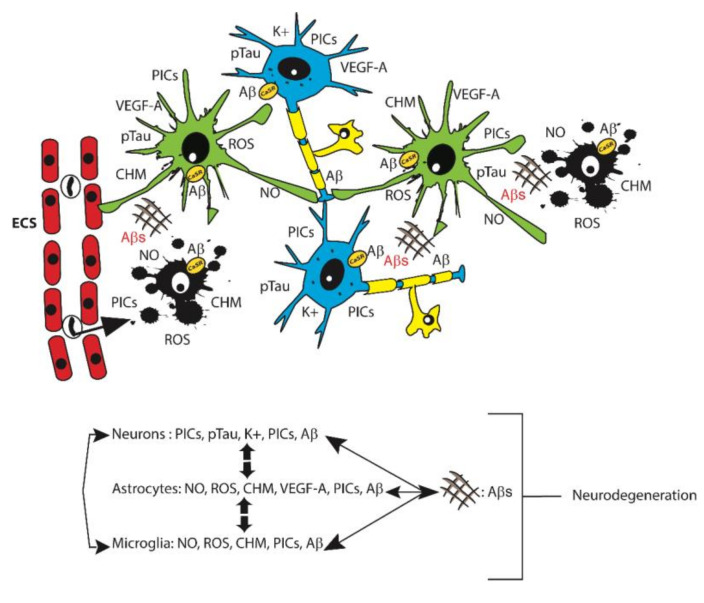
**Top**: A summary representation of the main cell types and proinflammatory factors each of them releases into the extracellular matrix in the course of AD. Neurons, blue. Astrocytes, green. Oligodendrocytes, yellow. Microglia, black. Endothelial cells (ECs), red. Monocytes, colorless. A black arrow indicates the migration of a monocyte into the nervous tissue. Senile plaques, ## Aβs. Most of the abbreviations as in the text. CHM, chemokines. PICs, proinflammatory cytokines. CaSR, calcium-sensing receptor. **Bottom**: Schematic diagram of the reciprocal interactions between the three main neural cell types involved in Alzheimer-related neuroinflammation. The bidirectional interaction with amyloid senile plaques is also indicated.

A still largely held view posits that in human AD the astrocytes behave as competent phagocytes tasked with cleaning up cellular debris and Aβ_42_ fibrils becoming overloaded with the latter in the process [63,64,65]. However, cortical nontumorigenic adult human astrocytes (NAHAs) exposed in vitro to fibrillary or soluble Aβ_25–35_, an Aβ_42_ proxy, de novo produce, accumulate, and release surpluses of Aβ_42_-os just like neurons do, while simultaneously decreasing their release of neurotrophic and neuroprotective soluble amyloid precursor protein-α (sAPP-α). Therefore, given their high numbers, the astrocytes can directly contribute to the amyloid brain overload proper of AD [33,66]. In addition, each astrocyte envelops with its mobile processes the so-called tripartite synapses of several neurons [67,68]. In addition, distinct astrocytes envelop with their processes the synapses of a single neuron. In this way, extended groups of neurons are functionally joined to regulatory astrocytes, while the latter are reciprocally interconnected via gap junctions that allow for the quick diffusion of Ca^2+^ waves [69]. Regulatory astrocytes also promote the formation and stabilization of their neurons’ synapses by mopping up released K^+^ ions and glutamate. They modulate the neuronal release of neurotransmitters by secreting their own gliotransmitters [70] and via Ca^2+^ releases and uptakes during their Ca^2+^ waves [69]. Importantly, astrocytes’ processes also envelope cerebral microvessels and affect the local blood flow and oxygen supply to support the functions of their metabolically dependent neurons [70]. An age-related acute or chronic local perfusion deficit and consequent brain tissue hypoxia can cause the accumulation and release of newly produced neurotoxic sAβ-os from both neurons and astrocytes [71]. This engenders vicious feed-forward cycles that stimulate the de novo surplus production and release of further amounts of sAβ-os, p-Tau-os, NO, VEGF-A, proinflammatory cytokines and chemokines, and other neurotoxic agents (Figure 1) [13,15,33,66].

But how can sAβ-os bring about such self-propagating tissue damage? And how might all the involved harming mechanisms be effectively neutralized?

## 3. Danger-Sensing/Pattern Recognition Receptors (PRRs)

The concept generally accepted in recent years is that the interactions between sAβ-os and a number of multiligand cellular receptors grouped under the denomination of “Pattern Recognition Receptors” (PRRs) may mediate via their intracellular signaling pathways all the noxious effects proper of AD’s neuropathology, including the associated neuroinflammation, particularly involving activated microglia and astrocytes [72] and the other CNS cell types too. Therefore, this review will focus on the receptorial interactions of Aβs that feasibly advance AD’s progression, particularly about microglia and astrocytes, without neglecting wherever opportune the other CNS cell types.

The evolutionarily highly conserved PRRs are integral parts of the innate immune system. The major PRRs families incorporate scavenger receptors (SRs; e.g., RAGE), Toll-like receptors (TLRs), NOD-like receptors (NLRs), AIM2-like receptors (ALRs), RIG-like receptors (RLRs), CLEC-2 receptors [73], and the calcium-sensing receptor (CaSR) [74]. The sensors of each PRR family pick out a heterogeneous set of specific exogenous pathogen-associated or endogenous damage-associated molecular patterns (PAMPs or DAMPs, respectively) belonging to complex products released from microorganisms or from the different compartments of injured cells or accumulating in the extracellular matrix (ECM). These PAMP•PRR or DAMP•PRR interactions drive the formation of several multicomponent protein signaling platforms, the inflammasomes, evoking the activation of caspase-1 and hence the maturation of proinflammatory cytokines precursors (pro-IL-1β and pro-IL-18), thereby inducing septic or aseptic inflammatory responses. The latter enhance PAMPs or DAMPs phagocytosis, eliciting synaptic apoptosis (synaptosis), and a caspase-1-dependent inflammatory cell death or pyroptosis [75], and sometimes also promoting the resolution of inflammation. DAMPs contribute to the host’s defense by interacting with PRRs, such as RAGE, TLRs, and inflammasomes, and activating the innate immune system [76,77]. However, when dysregulated and/or persistent the same DAMPs•PRRs or PAMPs•PRRs interactions can unphysiologically promote chronic inflammatory responses that cause the development and/or advance the progression of several human inflammatory diseases [76,78,79,80,81,82].

In the following paragraphs we shall review the PRRs families known to be involved in experimental or clinical AD.

### 3.1. Scavenger Receptors (SRs)

SRs are cell surface receptors that typically bind multiple ligands and promote the removal of non-self (PAMPs) or altered self (DAMPs) targets. SRs function through mechanisms including endocytosis, phagocytosis, adhesion, and intracellular signaling, which lead to the elimination of the degraded or harmful PAMPs or DAMPs. SRs mediate the uptake of fAβs in vitro [83]. SRs are widely distributed in Nature; their nomenclature and classifications have been revised several times [84]. The present discussion only refers to mammalian SRs.

#### 3.1.1. Triggering Receptor Expressed on Myeloid Cells 2 (TREM2) and 1 (TREM1)

TREM2 is a type I transmembrane cell surface receptor expressed by cells of the myeloid lineage, i.e., monocytes, macrophages, osteoclasts, CNS microglia, and dendritic cells [85,86]. It is a glycoprotein with an immunoglobulin (Ig)-like extracellular domain, a transmembrane domain, and a small cytoplasmic tail. In neural cells, TREM2 has no intracellular signaling system; hence it signals through its transmembrane binding partner, the 12 kDa DNAX activating protein (DAP12 aka TYROBP) that is endowed with an immunoreceptor tyrosine-based activation motif (ITAM) [87]. TREM2′s ligands are multiple, both exogenous PAMPs (from Gram-negative and Gram-positive bacteria, yeasts, and viruses) and endogenous DAMPs (usually polyanionic ligands and phospholipids) [88,89]. TREM2 serves as an anti-inflammatory receptor, negatively regulating the innate immune response via PI3K/NF-κB signaling [89]. Several studies have shown that TREM2 function is crucial for microglial adhesion to and phagocytosis of Aβs, and for neuroinflammation control [90,91,92,93,94]. Experimentally exploiting *TREM2* knockout mice disclosed that the direct targets of TREM2 signaling are IL-1β, TNF-α, secreted phosphoprotein-1, and C-C Motif Chemokine Ligand 2 (CCL2); and transcriptome analysis uncovered that TREM2 plays relevant roles in microglia chemotaxis, mobility, migration, and proliferation [95]. Moreover, *TREM2* knockout deeply affected microglia’s metabolism via the mammalian target of rapamycin (mTOR) pathway causing ATP depletion, cell stress, and cell death. Thus, TREM2 may crucially modulate transitions between microglial pro-inflammatory (M1) and anti-inflammatory or homeostatic (M2) phenotypes [96]. Regarding brain neurodegenerative diseases, AD included, abundant evidences show that *TREM2* knockout or hemizygous *TREM2* or deficient *DAP12/*TYROBP significantly alters microglia’s behavior, intensifying its proinflammatory cytokines production, neuroinflammatory responses, and neuronal debris clearing activity [90,97,98]. Importantly, *TREM2* haploinsufficiency or *TREM2* knockout strongly reduces or no longer allows, respectively, microglial proliferation and microgliosis to occur around brain Aβ plaques; contrariwise, it lessens or prevents microglial activation and induces microglial apoptosis in APP_swe_/PS1-21 and 5XFAD AD-model mice [90,99]. Consequently, *TREM2* knockout microglia does not enfold and compact Aβ plaques and does not form an isolating barrier preventing the plaques branching diffusion, the Aβ subspecies plaque content alteration, and the Aβ-related damage to dystrophic axons and dendrites, synaptic connections, and neurons in AD’s early stages [95,99]. Moreover, TREM2 function may be even more complex. In fact, in young AD model mice TREM2 knockout beneficially affected CD45^high^ myeloid cells; but later, once these CD45^high^ cells had died, TREM2 knockout became harmful by altering the proliferation, functions, and phenotypes of CD45^low^ myeloid cells [100]. Conversely, TREM2 overexpression curtailed the levels of proinflammatory cytokines and the activity of the two main Tau protein kinases, i.e., glycogen synthase kinase-3β (GSK-3β) and cyclin-dependent kinase-5 (CDK-5), thereby lowering neurotoxic p-Tau levels and neuronal deaths in P301S Tau-transgenic mice [93,101]. However, TREM2 overexpression did not improve the neuropathology and cognitive impairment in aging APPswe/PS1dE9 AD-model mice [102]. Interestingly, apolipoprotein E (APOE)•TREM2 complexes promoted Aβs uptake by microglia [103] and caused microglial to shift from homeostatic (M1) to neurodegenerative (M2) phenotype [104]. Blocking APOE•TREM2 signaling rescued microglia’s homeostatic phenotype including its tolerogenic function in AD-model mice [103,104]. In addition, Keren-Shaul et al. [105] reported a new microglia type linked to neurodegeneration (disease-associated microglia or DAM) and endowed with intracellular/phagocytic Aβ particles found in both mice and human AD brain slices. The transition from homeostatic microglia expressing Cx3cr1, P2ry12, and Tmem119 genes to DAM occurred in two steps. The TREM2-independent first DAM activation involved the downregulation of checkpoints. Next, a TREM2-dependent program followed including the upregulation of both TREM2 and lipoprotein lipase paralleled by increases in phagocytic activity and lipid metabolism, respectively. In fact, TREM2 perceives a spectrum of anionic and zwitterionic lipid ligands binding fAβs on the surface membranes of damaged neurons [90]. The precise mechanism operated by APOE•TREM2 signaling is unclear, but its importance is stressed by the fact that this unique microglia-type might hinder neurodegeneration.

Interestingly, γ-secretase-dependent intramembranous proteolytic cleavage can shed the soluble (s)TREM2 extracellular domain [106]. sTREM2 may also result from alternative splicing [107]. The cerebrospinal fluid (CSF)’s sTREM2 levels raise with ageing and become further heightened in AD patients, correlating with the CSF levels of total Tau and p-Tau proteins, but not with CSF levels of Aβ_42_ [108,109]. Moreover, sTREM2 boosted PI3K/Akt-dependently microglial survival and NF-κB-dependently kindled proinflammatory cytokines production. Importantly, in both wild-type and TREM2 knockout mice hippocampal delivery of sTREM2 upregulated microglia’s proinflammatory cytokines expression while changing the morphology and promoting the survival of the microglia [110]. These observations supported the hypothesis that CSF’s sTREM2 might be a marker of Tau dysfunction and of neuroinflammation.

Furthermore, little is known about TREM2 impact on intracellular Tau pathology. TREM2 knockout heightened neuronal Tau pathology and widely activated stress kinases, e.g., ERK1/2 and JNK, in a tauopathy-model mouse [111]. In addition, cerebrospinal fluid (CSF) analysis revealed that a rare TREM2 p.R47H, rs75932628 variant associates with an increased risk of AD in people of European descent [112]. European AD patients carrying such a risk allele exhibited elevated levels of total Tau, but unchanged levels of Aβ42, in their CSF [113]. Notably, the R47H, rs75932628 variant hampers TREM2 sensing of lipid ligands [90]. However, the TREM2 p.R47H rs75932628 variant is absent from Korean and Chinese people [114].

Finally, a few heterozygous TREM2 variants are associated with other neurodegenerative diseases including late-onset AD [115,116].

Monocytes and microglia also express the triggering receptor on myeloid cells 1 (TREM1) that affects Aβs phagocytosis by signaling through TYRO protein tyrosine kinase binding protein. Jiang et al. [117] showed that TREM1 knockout in microglia evoked increases in Aβ_1-42_ and total Aβs load in the brains of APP/PSEN1 AD-model mice. Conversely, triggering TREM1 signaling via an agonist antibody or overexpressing TREM1 in mouse microglia improved Aβ neuropathology and AD-related spatial cognitive deficits. Interestingly, an intensified Aβ neuropathology occurred in elderly humans carrying the TREM1 intronic variant rs6910730G. Moreover, the rs6910730G variant hampered Aβ phagocytosis by reducing TREM1 expression in human monocytes. These results supported a role for TREM1 in Aβ clearance by microglia worth further investigations.

#### 3.1.2. Receptor for Advanced Glycation Endproducts (RAGE)

RAGE belongs to the Immunoglobulin Superfamily and is found on the surface of various immune cells; most of its ligands are mainly secreted by immune cells, including macrophages and dendritic cells; therefore, the major roles played by RAGE relate to inflammation [118]. Neurons, astrocytes, microglia, and vascular cells (pericytes, smooth muscle cells, and endothelial cells) express RAGE in the CNS. Nucleic acids, lipids, and proteins undergo glycations intracellularly within cellular organelles and the cytosol, and within the extracellular matrix (ECM) thus becoming RAGE ligands (AGEs). The expression of both RAGE and its ligands is intense during the embryonic development when they favor the survival of neurons; thereafter, their expression is downregulated and remains at low levels up to old age [119].

Under physiological conditions, anti-glycation defenses efficiently clean off glycated lipids and proteins, while excision repair cuts off glycated nucleotides. AGEs accumulate in aging people and in the course of chronic diseases—such as AD, diabetes, atherosclerosis, arthritis, infections, cancers, and cardiovascular ailments—that are due to various known causes, such as UV light exposure, oxidative stress, malnutrition, epigenetic factors, and to as yet undefined agents. Under such conditions, RAGE and its ligands can be abnormally upregulated in both the CNS and peripheral tissues [120,121]. Accumulating AGEs of various kinds damage cell membranes, cross-link proteins, hamper the function of biological pathways, promote DNA mutations, and curtail mitochondrial ATP production. Recently Ibrahim et al. [122] put forward the view that RAGE is one of the PRR (see also below) interacting in this role with members of the Toll-like receptors (TLRs) family.

**Figure 2:**
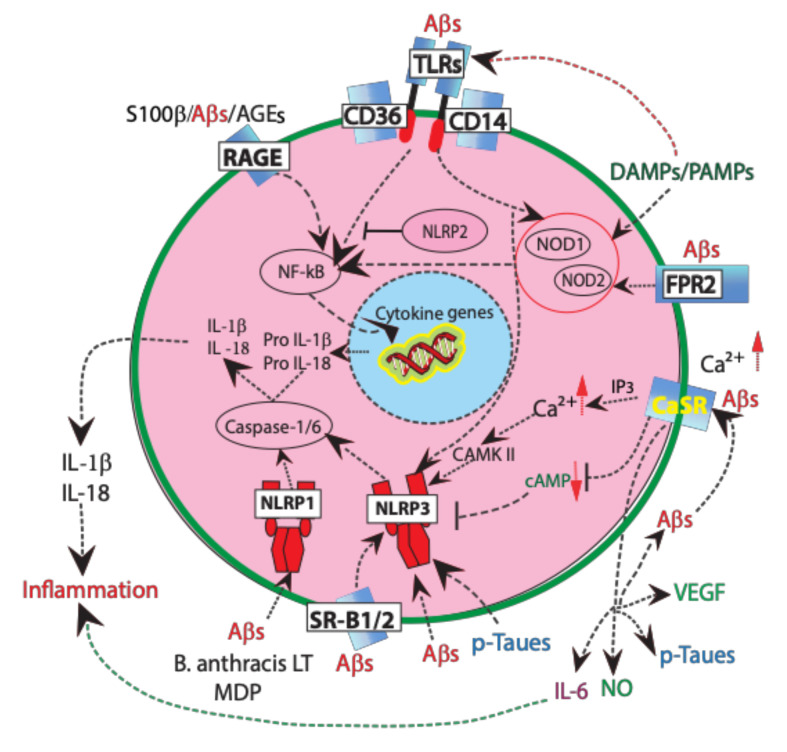
A schematic representation of some of the main PRRs and their signaling pathways involved in AD’s neuroinflammation. The cell represented could belong to any neural cell type. Aβs, soluble and/or fibrillar amyloid-β peptides; FPR2, formyl peptide receptor 2; IP3, inositol triphosphate; LT, lethal toxin; ↑, upregulation; ↓, downregulation. For further details and abbreviations see the text.

The RAGE’s cytoplasmic domain interacts with the formin homology 1 (FH1) domain of Diaphanous 1 (DIAPH1), a formin acting as the crucial point of ligand•RAGE-stimulated signal transduction [123]. On the opposite side, RAGE extracellular domain binds several AGEs, including Aβs, S100β proteins, high-mobility group box B1 (HMGB1) protein, heat shock (HS) proteins, and other endogenous danger-associated molecular patterns (DAMPs) that damaged or dying cells release in non-infectious conditions [124,125] (Figure 2).

The expression of these various DAMPs increases in course of chronic inflammatory ailments [126]. Serum levels of the soluble forms of both RAGE and HMGB1 are higher than normal in AD patients and correlate with their respective brain amyloid loads [127]. Unsurprisingly, some authors proposed to target the blockage of RAGE as an anti-AD therapeutic intervention [128,129,130]. As an example, metformin, an activator of AMP-activated protein kinase (AMPK), protects against diabetic AGE-induced harm in human neural stem cells (hNSCs). In fact, hNSCs exposed to AGEs exhibited an elevated expression of quite several proinflammatory cytokines, such as IL-1α, IL-1β, IL-2, IL-6, IL-12, and TNF-α, and concurrently a remarkable reduction in cell viability. Co-treatment with metformin, a first-line oral antidiabetic drug, abrogated the AGE-mediated noxious effects on hNSCs. In addition, metformin rescued the transcript and protein expression levels of acetyl-CoA carboxylase (ACC) and of inhibitory kappa B kinase (IKK) in AGE-treated hNSCs. Thus, metformin prevented the AGE-mediated proinflammatory increases in NF-κB mRNA and protein levels in hNSCs [131]. Therefore, the thus proven RAGE/NF-κB axis role in neuroinflammation has supported the “Nonenzymatic Glycosylation Theory of Aging”, suggesting both an AGEs key role in ageing-induced cognitive decline and a potential benefit of nutraceuticals in preventing neuroinflammation and AD [132].

Interestingly, while increasingly forming AGE•RAGE complexes upregulate RAGE expression. Therefore, the intensity of RAGE signals progressively increases as AGEs keep accumulating [133]. As just mentioned, the AGE•RAGE complexes mainly activate the NF-κB intracellular signaling pathway (Figure 2). NF-κB next enters the nucleus and turns on its target genes, including those encoding for several cytokines involved in adaptive or in innate immunity [134,135]. NF-κB targets and downregulates the expression of anti-apoptotic genes, such as those encoding BCL proteins, thereby working against cell survival [136]. Moreover, NF-κB binds glyoxalase (Glo)-1 and curbs its activity inhibiting AGEs production [137]. NF-κB also increases the expression and release of various proinflammatory cytokines, e.g., IL-1β, IL-6, IL-18, and TNF-α from cultured rodent astrocytes. By acting together with NO surpluses, the latter cytokines transform normal astrocytes into reactive astrocytes that promote the onset and/or progression of neurodegenerative disorders [138,139,140]. In addition, RAGE stimulation increases the activities of MAPKs, such as ERK, p38, and JNK, which induce cells to proliferate [141]. As an example of the complexities of RAGE-mediated signaling, let us briefly consider the effects of S100β•RAGE complexes. They did activate three distinct pathways in astrocytes. i.e., the RAGE/Rac-1/Cdc42, the RAGE/ERK/Akt; and/or the RAGE/NF-κB one. The latter next brought about several features proper of reactive astrogliosis, such as hypertrophy, proliferation, and migration. The same astrocytes also acquired a proinflammatory phenotype by expressing IL-1β (the result of inflammasome signaling activation), NO synthase (NOS)-2, and Toll-like receptor (TLR)-2. Finally, the astrocytes also induced an oxygen-glucose deprivation that furthered neurons’ death. Altogether, these findings demonstrated that S100β•RAGE complexes concurrently turn on several RAGE-dependent signaling pathways that profoundly change the astrocytes’ physiological phenotype into a proinflammatory and proneurodegenerative one [142]. These dysfunctional reactive astrocytes keep oversecreting surpluses of cytokines/chemokines and other proinflammatory mediators, which hinder neuronal glutamate uptake, promote synapses apoptosis (or synaptosis), and advance AD’s neuropathology spread, neurons’ death, and progressive cognitive deficits [143,144].

Regarding the effects on microglia, the persisting overrelease of proinflammatory cytokines partly prompted by AGE•RAGE signaling hampered microglia’s ability to clear the foci of synaptic apoptosis (or synaptosis) occurring without any neuronal death. Synaptosis pruning is mediated on one side by neuronal integrin α_ν_β_3_/Tyro3, Axl, and Mer (TAM) receptors plus bridging molecules and on the other side by the microglial PRR C3 receptor. Both these receptor types are necessary for the recognition of focal compartments of synaptic apoptosis exposing phosphatidylserine (PS) and tagged by C1q/C3 complement components [144]. Moreover, AGE•RAGE signaling also undermined microglia’s Aβ-clearing activity. Such microglia dysfunctions can advance brain Aβs load and neuroinflammation.

Remarkably, the neurons of aged 3xTg-AD model mice exhibited an increased RAGE-positive immunoreactivity, whereas glial cells were much less immunoreactive, save for the RAGE-expressing astrocytes in the hippocampal CA1 area. In addition, RAGE-positive immunoreactivity co-localized with intracellular but not with extracellular APP/Aβ complexes. In histological sections of human AD brains, most astrocytes possessed both AGE-positive and RAGE-positive cytoplasmic granules, whose distribution was superimposable. The Aβ-positive granules were less frequent but colocalized with AGE-positive and RAGE-positive granules in discrete cytoplasmic districts of each astrocyte [145]. Notably, Aβ plaques form only after the overproduction of ROS in vivo. In neurons cultured in vitro, Aβ•RAGE signaling increased ROS generation from NADPH oxidase, triggered the downstream phosphorylation of ERK-1/2 and p38 MAPK and of cytosolic phospholipase A₂ (cPLA₂), while inhibited long-term potentiation (LTP) in entorhinal cortex slices [146]. In turn, the accumulating ROS increased the synthesis of Aβs, of DAMPs such as HMGB1, and of S100β proteins, which are all RAGE ligands, thus bringing about an additional ROS overproduction further advancing AD’s neuropathology [147]. Interestingly, also the expression of human Tau co-localized with RAGE immunoreactivity in the hippocampal CA1 area of aged 3xTg-AD model mice [148]. By activating GSK-3β and p38 MAPK the AGE•RAGE-dependent signaling at least in part upregulated APP’s cleavage by BACE1/β-secretase and by γ-secretase leading to the generation of Aβs [149]. According to Batkulwar et al. [150] the AGE•RAGE signaling stimulated Aβ_1-42_ and p-Tau synthesis via increases in expression and activity of cathepsin B and asparagine endopeptidase (AEP), respectively. In human AD brains, the increases of AGEs concurred with heightened levels RAGE, Aβ_1-42_, p-Tau, and cathepsin B, thus linking AGEs to AD neuropathology. Of note, repeated injections of anti-RAGE antibody into the hippocampus reduced local Aβs and p-Tau^Ser−202^ accumulation, Akt/mTOR signaling, Ca^2+^-binding adapter molecule-1, and GFAP expression, and concurrently rescued behavioral deficits associated with cognitive decline [129].

Moreover, in brain/cerebral endothelial cells (BECs/CECs) RAGE neutralization via a specific antibody effectively blocked the Aβ-induced activation of ERK and JNK signaling that otherwise led to an increased matrix metalloproteinase-2 (MMP-2) expression exacerbating vascular inflammatory stress in both cerebral amyloid angiopathy (CAA) and AD [151]. Aβ₄₂ competed with the Ab(RAGE) (or anti-RAGE antibody) for RAGE binding on BECs/CECs and primary astrocytes plasma membranes. In both these cell types, Ab(RAGE) did abrogate the Aβ₄₂-evoked ROS overproduction and the activating co-localization of the NADPH oxidase cytosolic (p47-phox) subunits and the membrane (gp91-phox) subunits. Moreover, agents like an Ab(RAGE) or an NADPH oxidase inhibitor or a ROS scavenger hampered the Aβ₄₂-induced ERK1/2 and cytosolic PLA₂ phosphorylation in BECs/CECs. At the same time, only Ab(RAGE) but neither an NADPH oxidase inhibitor nor a ROS scavenger inhibited the ERK1/2 pathway and cPLA₂ phosphorylation in primary astrocytes. Finally, the infusion of a dominant-negative soluble RAGE form into the same animals decreased the brain’s Aβs load while improving synaptic transmission and hence learning and memory faculties [152,153].

#### 3.1.3. CD36 Superfamily SRs

CD36 superfamily SRs are PRRs distinguished between class A (i.e., SR-A, CD204, and MARCO [macrophage receptor with collagenous structure]) and class B (i.e., SR-B1, SR-B2/CD36, LIMP-2 [lysosomal integral membrane protein 2]/LGP85).

SR-As (aka macrophage scavenger receptors and cluster of differentiation 204 [CD204]) include five members, i.e., SR-A1-5. They are type II transmembrane proteins located at the cell surface. Functional SR-As have a homotrimeric structure consisting in two C-terminally different subunits that contain six functional domains [154]. SR-As can form complexes with at least 26 distinct classes of compounds including DAMPs like AGEs, apoptotic cells, ApoAI and ApoE, acetyl LDL, oxidized LDL (oxLDL), fAβs, etc.; and PAMPs such as human cytomegalovirus, crocidoiloite asbestos, silica, and more (see for further details and references, [155]). In mammalians, the most studied variant is SR-A1. Notably, SR-A3 is not inserted into the plasma membrane and hence does not partake in lipoprotein binding and uptake; yet it may act as a dominant negative controller of SR-A1 and SR-A2 (or MARCO) [156]. At variance with the other members, SR-A3 and SR-A4 are devoid of the highly conserved C-terminal cysteine-rich (CR) domain proper of the innate immune system and hence do not bind some DAMPs like ferritin [157]. SR-As roles are many and various, being involved in lipid metabolism and atherogenesis, but importantly also in innate immunity, host defense, inflammation, cardiac and cerebral ischemic injury, sepsis, and AD, playing sometimes a benign host-protective role, such as when binding components of Gram-positive or Gram-negative bacterial cell wall, but sometimes a noxious role, and sometimes a controversial role. Concerning AD, SR-As of astrocytes and microglia (i) bind fAβs and sAβ-os; (ii) induce Aβs phagocytosis; and (iii) activate intracellular MAPK signaling cascades, thus helping curtail the brain Aβs load and decreasing the release of proinflammatory cytokines [158,159]. In late-stage AD-model mice, SR-As levels diminished whereas those of inflammatory cytokines raised, thereby advancing AD progression [160]. Brain tissues from human AD patients exhibited a heightened SR-As expression in senile plaques and activated microglia [161].

SR-A2/MARCO is expressed in macrophages. It binds, and internalizes both DAMPs, like polyanions and ox-LDL, and apoptotic cells remnants. It also drives innate immune responses protecting against PAMPS including a wide variety of bacteria, e.g., *Neisseria meningitidis*, and of fungal pathogens, e.g., *Cryptococcus neoformans*. The induction of inflammation strictly requires that DAMPs or PAMPs are ligands of both SR-A2/MARCO and TLR-9 to allow NF-κB activation, the synthesis and release of proinflammatory cytokines, and the clearing of infectious agents. Like SR-A1, SR-A2/MARCO binds and endocytoses Aβs and may promote the progression of AD [162,163,164,165,166].

Multiligand SR-B1 acts physiologically as a specific receptor for high-density lipoprotein (HDL) and permits the selective nonendocytic influx of HDL-linked cholesteryl esters (CE) into cells by forming a hydrophobic transmembrane channel leaving the HDL on the outside [167]. SR-B1 function is important for steroidogenesis in the adrenals and ovary, which intensely express it. SR-B1 also mediates cholesterol efflux for the reverse cholesterol transport (RCT) from peripheral tissues to the liver via circulating HDL-CEs to be used for biliary acid synthesis and biliary cholesterol excretion [168,169]. By controlling cholesterol metabolism SR-B1 protects against atherosclerosis, myocardial infarction, and stroke [170,171]. Notably, SR-B1-mediated RCT is hindered by accumulating AGEs [172]. Macrophages bearing the HDL•SR-B1 complexes shift toward the antinflammatory (M2) phenotype via Akt activation; release increased amounts of anti-inflammatory cytokines IL-10 and transforming growth factor-beta (TGF-β); cut NF-kB activation down; and also mediate the removal of apoptotic cells (efferocytosis) via the Src/PI3K/Akt/Rac1 signaling pathway [173]. Being a multiligand receptor, SR-B1 also binds and internalizes several other unmodified and pathologically modified ligands, including liposoluble vitamins, carbohydrates, phospholipids, proteoglycans, ferritin, and pathogens e.g., hepatitis C virus, by cooperating with tetraspanin CD81, and the tight junction (TJ) protein claudin-1 (CLDN1) [168,174]. Recently Iram et al. [175] demonstrated that a link exists between SR-B1 and complement component 1q (C1q) in astrocytes isolated and cultured from aged 5xFAD model mice characterized by (i) a higher GFAP expression than the wild-type mice astrocytes; (ii) an impaired ability to uptake fAβs; and (iii) a reduced SR-B1 expression correlated with an impaired Aβ uptake. Preincubating exogenous C1q dose-dependently with Aβ_42_ and then adding C1q plus Aβ_42_ to old 5xFAD astrocyte cultures rescued the impaired Aβs uptake and clearance by the astrocytes. These results revealed that C1q works as an extracellular chaperone that cooperates with SR-B1 to recover astrocytes’ ability to uptake and clear Aβs. The perivascular macrophages associated with senile plaques also express the SR-B1 receptor. In J20 transgenic AD-model mice, SR-B1 acts as a go-between in perivascular macrophages responses to fAβs thus regulating both Aβ-related neuropathology and CAA. In comparison with wild-type J20 animals, a single SR-B1 allele inactivation in J20/SR-B1^+/−^ mice increased brain fAβs accumulation and CAA intensity thereby worsening deficits in memory and learning. Therefore, a reduced SR-B1 expression hampered the reaction to fAβs on the part of perivascular macrophages while aggravating the Aβ-related neuropathology and CAA in J20 AD-model mice [176].

PRR SR-B2, aka CD36, is a type II transmembrane glycoprotein. Like SR-B1, the SR-B2/CD36 receptor is ubiquitously expressed. It binds and endocytoses numerous ligands, including fAβs, long-chain fatty acids, oxidized low-density lipoproteins (oxLDLs), oxidized phospholipids (oxPLs), hexarelin, and thrombospondin-1. Besides, SR-B2/CD36 receptor also binds and internalizes PAMPs like apoptotic bodies, *Staphylococcus* and *Mycobacterium* cell wall components, and *Plasmodium falciparum*-infested erythrocytes [177]. Vascular macrophages’ SR-B2/CD36s bind and internalize oxLDLs, which by a feed-forward mechanism further intensify SR-B2/CD36 expression and oxLDLs uptake, resulting in the overproduction of ROS and proinflammatory chemokines that promote vascular inflammation and atherosclerosis [178]. Both microglia and BECs/CECs of AD patients express the SR-B2/CD36 receptor. The binding of fAβs to microglia-expressed SR-B2/CD36 receptors mediates H_2_O_2_ overproduction in human AD brains [179]. Moreover, SR-B2/CD36 binds soluble Aβs in vitro [160] and mediates microglial and macrophage responses to Aβs in mice [158]. Most important, the SR-B2/CD36 receptor is the primary regulator of NOD-like receptor (NLR) protein inflammasome 3 (NLRP3) signaling activation (see Figure 2 and further on) in AD, atherosclerosis, and type-2 diabetes. In fact, SR-B2/CD36 promotes the accrual and intra-lysosomal conversion of soluble monomeric Aβs to soluble Aβ-os/fibrillar Aβs and the consequent activation of the NLRP3 inflammasome signaling leading to IL-1β oversecretion [180]. Upon binding to Aβs, microglia’s SR-B2/CD36 receptors can form complexes with other cell surface receptors, i.e., CD47, α_6_β_1_-intergrin, TLR-4 and TLR-6. Such complexes further stimulate microglia’s production of proinflammatory mediators [181].

Microglia of normal adult human brain express neither SR-A nor SR-B receptors, while astrocytes express only SR-B1. However, astrocytes express both SR-B1 and SR-A and microglia SR-A receptors in Alzheimer’s brains. Astrocytes and microglia accumulate around senile plaques, and adhere to and engulf fAβs, while microglia also release ROS [182,183]. Neonatal rat microglia express immunodetectable SR-A1, SR-A2/MARCO, SR-B1, and RAGE. On the other hand, neonatal rat astrocytes express SR-B1 and SR-A2/MARCO. An adhesion assay revealed that astrocytes and microglia from neonatal rats adhered in a concentration-dependent way to surfaces overlaid with fAβs or nonfibrillar Aβs. Polyinosinic:polycytidylic acid (poly[*I*:*C*]) and fucoidan, known SR-A receptors ligands, inhibited the adhesion of microglia and astrocytes to fAβs and also hindered fAβs phagocytosis. In contrast, HDL, a SR-B receptors ligand, did not hamper glial cells’ adhesion to fAβs. Therefore, both microglia and astrocytes adhere to fAβs and nonfibrillar Aβs via a fucoidan-sensitive SR-A receptor that could be SR-A2/MARCO [184]. In cultured human astrocytes isolated from non-demented subjects and exposed to fAβs, the induced upregulation of neprilysin and SR-B1 gene expression was severely restricted by the presence of Aβ-associated proteins, chiefly APOE. In contrast, such neprilysin and SR-B1 changes no longer occurred in the astrocytes derived from long-standing AD patients due to a defective regulation of Aβ-clearing genes that could advance the progression of AD neuropathology. Under such circumstances the astrocytes became overburdened with Aβs and unable to support neurons’ metabolism and neurotransmitters recycling [185,186].

AD’s neuropathology also involves vascular amyloidosis and cerebral amyloid angiopathy CAA; both conditions cause local tissue hypoperfusion, hypoxia, and acidosis. Rodent glial cells exposed for 24-48 h to an acidified culture medium (pH 6.5–to–6.9) exhibited a reduced Aβs phagocytosis by astrocytes and a temporarily decreased one by microglia. In the astrocytes cultured at pH 6.5 SR-B1′s expression increased whereas SR-A2/MARCO expression decreased. Conversely, both SR-B1′s and SR-A2/MARCO’s expression surged while that of SR-B2/CD36 fell in microglia exposed to pH 6.5. In conclusion, the acidic environment can distinctively change scavenger receptors’ expression in astrocytes and microglia but enduringly decreased only the astrocytes’ Aβs clearing activity [187].

### 3.2. Toll-Like Receptors (TLRs)

TLRs belong to the PRRs superfamily. Humans have at least ten TLRs, whose expression occurs widely in the brain. Neurons and microglia predominantly express TLRs as compared to astrocytes and oligodendrocytes. TLRs respond to pathogens and cellular stressors (PAMPs) and to DAMPS. In general, TLRs response to pathogens has been more intensely investigated than that to DAMPs. However, regarding the progressive AD-related neuroinflammation the response of TLRs to DAMPs is more relevant. The TLRs ligand-binding extracellular domain is endowed with a variable number of N-terminal leucine-rich repeat (LRR) domains, which are involved in TLR dimerization [188,189,190]. The signaling of these receptors occurs through their carboxy-terminal intracellular tail that contains a Toll/IL-1 receptor (TIR) homology domain resulting in the recruitment of the cytoplasmic adaptor proteins MyD88 and TOLLIP (Toll interacting protein) and the activation of NF-κB-dependent genes, such as TNF-α, IL-1, IL-6, and IL-8. In AD neuropathology, TLRs are implicated in sensing and responding to the presence of different Aβ species. Several studies of postmortem brain tissues from AD patients and transgenic AD-model mice have found augmented expression levels of TLR-2, TLR-4, TLR-5, TLR-7 and TLR-9 as well as of the TLR co-receptor CD14, in microglia localized around senile plaques [190,191,192,193]. The detrimental impact of TLR-2 signaling in AD pathogenesis is due to the polarization of microglia toward a neatly proinflammatory profile (M1). In microglial cells the activation of TLR-2 by fAβs induces the production of proinflammatory mediators, such as inducible NOS-2, TNF-α, IL-1β, and IL-6 (Figure 2) [194]. Of note, TLR-2 knockout in APP_swe_/PS1dE9 mice decreases senile plaques load and mitigates neuronal damage [191,195]. Similarly, TLR-4, when stimulated by fAβs or sAβ-os, induces a strong release of the proinflammatory cytokines IL-1β, IL-6, TNF-α, CCL5, MIP-1α, and MCP-1 [196]. In response to Aβs TLR-2 and TLR-4 might also form complexes with other TLRs (e.g., TLR-4•TLR-6) and with cell surface receptors, e.g., CD14 and SR-B2/CD36, the latter acting as a co-receptor involved in the recognition of fAβs and sAβs. Sheedy et al. [180] showed that the TLR-4•TLR-6 complex and SR-B2/CD36 can harmfully cooperate and direct the activation of the NLRP3 inflammasome signaling pathway in AD-related neuroinflammation. Similarly, the CD14 association with the dimer TLR-2•TLR-4 forms a critical complex that activates microglia and promotes its ability to bind and phagocytose fAβs. Furthermore, the activation of TLR-9 in mice resulted in an increased Aβs uptake and clearance by microglia [197] and in a reactive astrogliosis [198].

### 3.3. NOD (Nucleotide-Binding Oligomerization Domain) Receptors and (NOD)-Like Leucine-Rich Repeat Receptors (NLRs) Inflammasomes

Inflammasomes are crucial cytoplasmic multimeric protein complexes or platforms that critically regulate the inflammatory responses of innate immunity. Among the most studied are the nucleotide binding oligomerization domain receptors 1 and 2 (aka NOD1 and NOD2) [199] and the NLRs (aka nucleotide-binding oligomerization domain [NOD]-like leucine-rich repeat) receptors, which belong to a family of intracellular PRRs initially denoted as cytoplasmic sensors of microbes. Inflammasomes are endowed with intracellular PRR receptors (or sensors) that recognize cell stress-linked DAMPs or pathogen-derived PAMPs. Every type of inflammasome has its own distinct receptors [200,201,202,203]. According to their N-terminus features, NLRs are classified into four subfamilies, i.e., NLRA, NLRB, NLRC, and NLRP. The NLRP family (NLRPs) interacts with the ASC (apoptosis-associated speck-like protein containing a CARD) adaptor protein endowed with an N-terminus PYRIN (aka DAPIN)-CARD domain activating pro-Caspase-1 [204]. In humans, NLRPs comprise 14 members, i.e., NLRP1, NLRP2, NLRP3, and so on. The PYRIN/DAPIN domain crucially determines inflammasome’s complex assembly and signal transduction [205]. The ligand specificity of each NLR receptors depends upon their leucine-rich repeats. Their variable N-terminus domains include caspase-1-binding CARD domain-endowed 4; absent in melanoma 2 (AIM2) protein; and IPAF (ICE protease-activating factor) that effect the activation of distinct biological pathways [206,207,208]. After interacting with PAMPs or DAMPs, PYRIN-PYRIN domains interactions elicit NLRs oligomerization and ASC recruitment [209]. Thereafter, CARD-CARD domains interactions lead to procaspase-1 binding to ASC activate the inflammasome [204]. The formation of complexes between PRR receptors and DAMPs or PAMPs induces the assembly and activates the inflammasomes’ signaling that triggers the NF-kB pathway, Caspase-1 activation, and cytokines IL-1β and IL-18 maturation [209,210,211] (Figure 2). Inflammasome activation can induce pyroptosis—an inflammatory form of caspase-1-mediated regulated cell death entailing an initial release through plasmalemmal breaks of the intracellular contents—in various CNS cell types [212,213]. Human neurons, astrocytes, and microglia all display robust NRLP3 inflammasome-associated responses [212]. No investigation concerned inflammasome activation and pyroptosis in human oligodendrocytes until recently when McKenzie et al. [214] observed it in vitro following an exposure to inflammatory stimuli, thus identifying a new mechanism of inflammatory demyelination. Neurons also express the NLRP1 and AIM2 inflammasomes [215], while astrocytes also possess the NLRC4 and NLRP2 ones [210,216] (Table 3).

The general view holds that PAMPs or DAMPs act as primers inducing the expression and maturation of both the NLR receptors precursors and of caspase-1. However, it is noteworthy that in the brain inflammasomes preexist as already assembled complexes that do not need the priming step thus giving out faster signals upon activation [220,221]. Therefore, inflammasomes assembly mechanisms may differ from one to another cell type. In response to harmful stimuli neurons, macroglia, and microglia increase the expression of inflammasome proteins, which are key modulators of the innate immune response triggered in AD [221,222,223]. The present understanding of the intricate transactions among so many inflammasomes and their regulatory mechanisms in different pathological conditions is limited: they will need further studies to be clarified.

#### 3.3.1. NLRP1 Inflammasome

The NLRP1 (aka NALP1) inflammasome was the first to be identified after spinal cord and brain ischemic injuries [224]. It comprises both an N-terminal PYRIN/DAPIN domain allowing the PYRIN-PYRIN-mediated NLR-nucleated oligomerization and the gathering of the PYRIN-CARD-endowed ASC adaptor proteins. A CARD-CARD interaction permits the binding of (pro)caspase-1 and the activation of inflammasome’s signaling. [204,209]. As a unique feature, the NLRP1 inflammasome also has a C-terminal CARD domain that can bind the (pro)caspase-1 CARD-domain independently of the ASC protein [207]. Another peculiar NLRP1′s feature is the FIIND (Function to Find) domain at its C-terminus, which must undergo autolytic cleavage at Ser^1213^ to allow NLRP1 activation [225]. Upon sensing proper stimuli, like muramyl dipeptide (MDP) and *Bacillus anthracis* lethal toxin (LT) [226], NLRP1′s oligomerization induces the aggregation of ASC adaptor proteins first into filaments and next into macromolecular specks promoting the CARD-CARD binding and autocatalytic activation of (pro)caspase-1. This leads to the de novo synthesis of IL-1β and IL-18, neuroinflammation, and neurons’ death by pyroptosis [213]. NLRP1 can assemble in neurons and glial cells (macroglia, and microglia/macrophages). Injecting a NLRP1-neutralizing antibody into postischemic mice hampered NLRP1 inflammasome activation, lowered proinflammatory cytokines levels, and safeguarded neurons viability. These findings proved NLRP1′s crucial role in neuroinflammation [220,227,228].

NLRP1 inflammasome signalling is involved in AD-related neuroinflammation. de Rivero Vaccari et al. [221] showed that once exposed to Aβ-os cultured neurons lost K^+^ ions via channel efflux. The consequently reduced intracellular [K^+^]_i_ powerfully activated the NLRP1 inflammasome signaling and caspase-1 and the latter cleaved the IL-1β and IL-18 precursors into their mature forms. In AD-model mice, Aβs activate the NLRP1 inflammasome in pyramidal neurons and oligodendrocytes through a yet not understood mechanism, eliciting the caspase-1-mediated IL-1β and IL-18 maturation and neurons’ death by pyroptosis, thus advancing cognitive decline. Similarly, serum-deprived (i.e., stressed) human neurons activated the NLRP1 (but not the NLRP3) inflammasome/caspase-1/caspase-6 pathway, which increased the Aβ_42_/Aβ_40_ ratio value and triggered neurons’ death and axonal degeneration. Remarkably, the NLRP1 inflammasome-expressing neurons were 25-to-30-fold more numerous in human AD brains than in non-AD brains [215,217]. Tan et al. [229] showed the upregulation of brain NLRP1 levels in B6C3-Tg (APP_Swe_/PS1dE9) AD-model mice. An in vivo knockdown of NLRP1 or (pro)caspase-1 in B6C3-Tg mouse brains significantly decreased neurons’ pyroptosis and mitigated cognitive impairment. The same authors demonstrated that Aβs administration increased the NLRP1-mediated caspase-1-dependent pyroptosis of cultured cortical neurons. Conversely, NLRP1 inflammasome expression levels remained unchanged in an experimental AD-like model induced via streptozotocin (STZ) injections in male Wistar rats [230]. Such discrepant results suggest that variations among distinct species and experimental stimuli do impact on the activation of specific inflammasomes in neuroinflammation during CNS injury and repair [231].

#### 3.3.2. NLRP2 Inflammasome

The multiprotein aggregate forming the brain NLRP2 inflammasome comprises NLRP2, the adaptor protein ASC, and (pro)caspase-1. In addition, NLRP2 inflammasome can team up with the P2X7 purinergic receptor and Pannexin 1 (Panx-1), a gap-junction channel releasing ATP. Humans express four isoforms of NLRP2 [232]. Interestingly, cultured human primary astrocytes exposed to exogenous ATP expressed an actively signaling NLRP2 inflammasome platform. Yet, untreated human astrocytes in vitro also released low amounts of IL-1β produced via the signaling of a preassembled NLRP2 inflammasome [210]. The administration of Probenecid, a blocker of Panx-1 gap junction channels, or of Brilliant Blue G (BBG), an inhibitor of the ATP-gated P2X7 purinergic receptor ion channel, and a siRNA-induced NLRP2 knockdown all decreased NLRP2 inflammasome activation and caspase-1 maturation/activation in ATP-treated human cortical astrocytes [210]. However, the function of NLRP2 in human astrocytes or in the brain in general is still mostly unknown. Interestingly, a misregulation of the *NLRP2* gene at an early stage of human fetal brain development may have resulted in a bipolar disorder [233].

Notably, in mice the NLRP2 gene-encoded mRNA and NLRP2, its translated protein, are essential for the early embryonic development [234]. A study from Sun et al. [235] reported that in the brain of wild-type C57BL/6J male mice NLRP2 protein had a low constitutive expression, which mostly took place in the astrocytes. Interestingly, NLRP2 expression significantly increased after an ischemic stroke in the brains of the same mice or in oxygen-glucose deprivation-exposed mouse astrocytes cultured in vitro. The authors suggested that NLRP2 may play an adverse, i.e., proapoptotic, role in the pathophysiology of brain stroke through mechanisms that remained undetermined. Furthermore, Kynurenine, a tryptophan metabolite, promoted NLRP2 expression and signaling in hippocampal astrocytes and did the same also in chronic mild stress (CMS)-induced depressive mice via an NF-κB-dependent regulation at the level of the *NLRP2* gene promoter [236].

As a further demonstration of the importance of the cell type or model used in relation to the function of specific inflammasomes, the results of NRLP2 knockout in THP-1 cells or of NRLP2 hyperexpression in HEK293T cells led Bruey et al. [237] to suggest that NLRP2 inhibits NF-kB activation by various stimuli at the IKK complex level and blocks the expression of *ICAM-1*, an NF-κB target gene.

#### 3.3.3. NLRP3 Inflammasome

The NLRP3 inflammasome (aka NLRP3-ASC or NOD-like receptor protein 3 [NACHT, LRR, and PYD domains-containing protein 3]), is involved in inflammatory and degenerative diseases typical of ageing that is in ailments characterized by the intra-tissue hoarding of peptides or crystals such as AD (Aβs), atherosclerosis (cholesterol), and gout (monosodium urate); in fibrotic diseases; and in metabolically stressful conditions, such as nonalcoholic hepatic steatosis, and type-2 diabetes [238]. Therefore, it is not surprising that NLRP3 is the most intensely studied inflammasome [239]. Typically, two initiating signals, one from a Toll-like receptor (TLR) agonist (“*priming”*) and another from a NOD-like receptor (NLR) agonist (previously endocytosed PAMPs or DAMPs), activate the NLRP3 inflammasome. Alternatively, exogenous ATP let out from dead cells; destabilized lysosomal releasing cathepsin B; phagocytosed protein polymers; reactive oxygen species (ROS); cardiolipin; oxidized DNA from damaged mitochondria [240,241,242]; K^+^ efflux or Ca^2+^ influx, independently of each other [243,244]; and reduced cAMP levels can all operate as NLRP3 activators [245,246] (Figure 2). Increases in cytosolic Ca^2+^ levels might be the upstream signal shared by all stimuli activating the NLRP3 inflammasome [74,247,248]. All stimuli activating the NLRP3 inflammasome converge toward the NF-κB pathway-mediated genetic transcription of NLRP3, pro-IL-1β and pro-IL-18; the latter once transformed by caspase-1 into mature IL-1β and IL-18 induce the neuroinflammation and cause neuronal death by pyroptosis [244]. The inactive NLRP3 moieties are restricted to the endoplasmic reticulum (ER) membranes, from which upon activation they migrate together the adaptor ASC protein to the perinuclear ER membranes and associated mitochondrial aggregates [240]. The dispersion of the *trans*-Golgi network (TGN) musters NLRP3 and ASC proteins together via an interaction with phosphatidylinositol-4-phosphate (PtdIns4P) [249]. *NLRP3* gene knockout or pharmacological blockage of NLRP3 activation have powerfully benefited several inflammatory diseases modeled in rodents [238]. A phosphodiesterase (PDE) inhibitor blocking cAMP catabolism or an adenylate cyclase activator (e.g., PGE2) or a modified (e.g., dibutyryl-) cAMP, all heightening the intracellular cAMP levels, can hamper the NLRP3 inflammasome activation and its noxious upshots [74,250].

The NLRP3 inflammasome plays a crucial role in AD-related neuroinflammation. Under normal conditions, the cytosolic NLRP3 inflammasome is inactive in both microglia and astrocytes. An exposure to fAβ_1–42_ followed by its phagocytosis with consequent lysosomal damage releasing cathepsin B induces the oligomerization and activation of the NLRP3 inflammasome resulting in the maturation of IL-1β [251]. On its own part, an activated NLRP3 inflammasome intensifies AD neuropathology in vivo [252]. Immunohistochemical studies showed that an increased expression of the NLRP3 inflammasome constituents, of (pro)caspase-1, and of the inflammasome activation products IL-1β and IL-18 colocalized with glia maturation factor (GMF), APOEε4, autophagic SQSTM1/p62- and LC3-positive vesicles, and the lysosomal marker LAMP1 in tissue samples from the temporal cerebral cortex of AD brains. The reactive astrocytes surrounding amyloid plaques overexpressed GMF, a highly conserved pro-inflammatory protein activating glial cells, thus advancing neuroinflammatory and neurodegenerative processes. By contrast, suppressing GMF expression alleviated the neurodegeneration. The results suggested that GM, intensified the NLRP3-driven neuroinflammation, thus hampering the autophagosomal pathway-mediated clearing of pathological Aβs aggregates [253,254,255]. Interestingly, these results implied that GMF might play a role not only in AD but also in other neurodegenerative diseases [256].

As previously mentioned, ASC is an adaptor and stabilizer of the NLRP3-ASC complex and a pivotal protein for its activation. That Aβs do activate astrocytes’ inflammasome(s) was shown by studies using *ASC*^+/−^ or *ASC*^−/−^ mouse models. ASC partial knockout downregulated the NLRP3 inflammasome activity; concurrently, an upregulated CCL3 chemokine release increased Aβs phagocytosis by lipopolysaccharide (LPS)-primed primary astrocytes from 5xFAD newborn mice. Moreover, the ASC partial knockout reduced the brain amyloid load in 7–8-month-old mice, which correlated with an increased *CCL3* gene expression and an improvement of spatial reference memory [257].

Aβ fibrils too can specifically activate the NLRP3 inflammasomes in the microglia of APP/PS1 AD-model mice. An experimentally induced NLRP3 inflammasome downregulation in the same mice shifted microglia polarization toward a beneficial M2 (or arginase-1-positive or homeostatic) phenotype, and concurrently depleted the brain amyloid load. Moreover, an intensely active IL-1β-producing caspase-1 was detected in human mild cognitive impairment (MCI) brains and in frankly symptomatic AD brains. Hence, NLRP3 inflammasome signaling activation appears to play a specific role in the microglia-mediated persistent neuroinflammation of AD [251,252]. Moreover, a study from Saresella et al. [258] proved the activation of NLRP1 and NLRP3 inflammasomes in both mild cognitive impairment (MCI) and late-stage AD patients. However, functional inflammasomes were not yet working in the MCI patients. But the concurrent activation of NLRP1 and NLRP3 inflammasomes would intensify the neuroinflammation in late-stage AD patients. Drugs such as Dihydromyricetin [259] or MCC950 [260] blocking NLRP3 inflammasome signaling promoted the clearance of Aβ, decreased the fraction of M1 activated microglia in the hippocampus and cerebral cortex, and ameliorated memory and cognitive deficits in APP/PS1 AD-model mice. Moreover, the endogenous protease inhibitor α1-antitrypsin (A1AT) reduced the Aβ_1–42_-elicited NLRP3 inflammasome activation in primary cortical astrocytes from BALB/c mice [261].

Besides Aβ, p-Tau protein is the other main driver of AD. The causal relationship (if any) between p-Taues and inflammasome activation remained unknown until Stancu et al. [262] showed that following microglial uptake and lysosomal sorting, prion-like Tau seeds activated the NLRP3 inflammasome signaling. Moreover, the chronic intraventricular administration of the NLRP3 inhibitor MCC950 significantly thwarted the neuropathology elicited by exogenous Tau seeds. Concurrently, the suppression of NLRP3 inflammasome function diminished p-Tau levels and aggregation by restraining the activity of Tau kinases while increasing that of p-Tau phosphatases [263]. Thus, a therapeutic approach targeting the NLRP3 inflammasome might affect the three main drivers of AD.

Moreover, Murphy et al. [264] showed that Aβ-treated primary rat glial cultures increased their cytosolic cathepsin activity, assembled the NLRP3 inflammasome, activated caspase-1, and released mature IL-1β.

Finally, using wild-type and double-stranded RNA-activated protein kinase (PKR) knockout mouse macrophages Lu et al. [265] showed that PKR was required for the direct physical interaction with and activation not only of the NLRP3, but also of NLRC4 and AIM-2 inflammasomes and consequently increased IL-1β production. However, using LPS-treated *PKR* knockout bone marrow-derived macrophages from different mouse strains He et al. [266] reported that PKR was critical for nitric oxide synthase-2 (NOS-2) induction but dispensable for the caspase-1 activation, cleavage of pro-IL-1β and pro-IL-18, and IL-1β release elicited by stimuli activating NLRP3, NLRC4 and AIM2. Altogether these divergent data indicate that the results reported about inflammasomes’ activation could be heavily conditioned by the animal species or strain investigated [231].

#### 3.3.4. AIM2-Like Receptors

AIM2 (absent in melanoma 2) protein is one of the most well-characterized AIM2-like receptors, also called PYHIN proteins, first characterized as a family of IFN-inducible proteins. It is a DNA-binding sensor located in the cytosol [267]. AIM2 forms filamentous signaling inflammasome complexes including ASC and caspase-1. Cytoplasmic long dsDNAs released from either infecting bacterial or viral pathogens or from damaged nuclear and/or mitochondrial DNA induce the assembly of the AIM2-ASC inflammasomes [268]. AIM2 is by far the dominant PRR sensor expressed in healthy mouse brains. However, in AD-model mice its role is still controversial. Wu et al. [269] reported that Aβs deposition and microglial cells activation in the cerebral cortex and hippocampus were less intense in AIM2 knockout 5XFAD mice than were in wild-type mice. Notably, via unknown mechanisms AIM2 deletion upregulated IL-6 and IL-18 expression, but neither changed IL-1 expression nor improved cognition in 5XFAD-model mice. These results also implied that different inflammasomes would play distinct roles in divers physiological and/or pathological circumstances or animal models.

#### 3.3.5. NLRC4 Inflammasome

NLRC4 is an evolutionarily conserved inflammasomal component of the innate immune system uniquely lacking the PYRIN domain. NAIPs (neuronal apoptosis inhibitor proteins) are a family of sensors binding PAMPs (e.g., flagellin) from several pathogenic bacteria to modulate the NLRC4 inflammasome activation [270]. The latter entails NLRC4 oligomerization and CARD-CARD interaction with Caspase-1 to induce pyroptosis in macrophages [75,205,271]. On the other hand, the association between NLRC4 and ASC leads to increases in cytokine production [272]. Phosphorylation by an unidentified protein kinase C (PKC) isoform is crucial for NLRC4 inflammasome activation [273].

Humans only have a single NAIP gene. Interestingly, Christie et al. [274] showed that NAIP-1 protein levels had decreased in the brains of fully developed AD cases as compared to those proper of the brains of MCI and control cases. In parallel, the levels of paired helical filament-1 (PHF-1) protein, a marker of Tau protein NFTs, were heightened in the same AD cases. The authors suggested that these changes increased the neurons’ risk to develop NFTs and die by apoptosis. Conversely, Lesné et al. [275] reported that neurotrophin-3 (NT-3) up-regulated the expression of NAIP-1, which inhibited Aβ-driven apoptosis in primary cultures of cortical neurons by restraining the activation of caspase-3, caspase-8, and caspase-9. Moreover, These NAIP-1′s protective effects required the activation of Akt and PI3K.

Notably, Down’s syndrome (DS) entails an early AD-like neurodegeneration sustained by an intensified neuronal apoptosis which causes the typical mental deficits. By using Western immunoblotting Seidl et al. [276] demonstrated that as compared to control subjects the levels of NAIP-like immunoreactivity are significantly lower in the parietal and occipital cerebral cortex in DS and in the frontal and occipital cortex in AD. Therefore, decreased levels of the neuroprotective NAIP may underlie the neurodegeneration occurring in both DS and AD.

Yet, the story may be even more complex than expected. Saadi et al. [230] used streptozotocin (STZ) injected male Wistar rats as experimental AD-like models and determined the expression of genes implicated in the inflammasome complex, such as *NLRP1*, *NLRP3*, *NLRC4*, *AIM2*, *ASC* or *PYCARD*, *IL-1β*, *IL-18,* and *CASPASE-1*. The authors detected in the STZ-treated animals significant surges only in NLRC4, ASC, and IL-1β expression levels with respect to the controls. In addition, the number of cells expressing caspase-1, IL-1β, and p-Tau proteins remarkably increased in the hippocampus of STZ-treated rats. The authors suggested the pathogenetic involvement of NLRC4 in AD and its potential therapeutic targeting. Freeman et al. [216] reached similar conclusions by using lysophosphatidylcholine (LPC), an agent linked to neurodegeneration and demyelination, which activated NLRP3 and NLRC4 inflammasomes in astrocytes and microglia. Therefore, NLRP3 and NLRC4 inflammasomes should act as important inducers of neuroinflammatory astrogliosis and microgliosis in AD, and in multiple sclerosis and stroke too.

#### 3.3.6. IPAF Inflammasome

For the sake of completeness, one must mention here the IPAF aka ICE protease-activating factor-apoptosis-associated speck-like protein containing a caspase activation and recruitment domains (CARD) (ASC) inflammasome. Liu and Chan [277] showed that palmitic acid activates the IPAF inflammasome in primary astrocytes causing the production of mature IL-1β. In addition, IPAF silencing suppressed the release of IL-1β from astrocytes, which reduced Aβ_42_ production on the part of primary neurons. Of note, Liu and Chan [277] also showed the overexpression of IPAF and ASC in the brain tissue of a subgroup of sporadic AD patients, which indicated the IPAF-ASC involvement in AD-related neuroinflammation and the potential usefulness of their therapeutic targeting in AD.

A later study revealed that the ER stress-associated transcription factor, C/EBP homologous protein (CHOP), played a critical role along a CHOP/NF-κB signaling pathway mediating the palmitate-elicited up-regulation of BACE1/β-secretase expression and of Aβ_42_ synthesis in astrocytes [278].

#### 3.3.7. NOD Inflammasomes

Chen and coworkers. [279] reported that intracellular NOD2 receptor cooperates with TLR-2 to increase the expression of formyl peptide receptor 2 (FPR2), which binds Aβ_1-42_ and signals via G proteins, in mouse microglial cells. These data indicate that TLR-2 and NOD2 may play a relevant role in neuroinflammation and AD. On the other hand, the intraperitoneal administration (10 mg Kg^−1^ once or twice weekly) for three or six months of muramyl dipeptide (MDP), a NOD2 receptor ligand, to 3-month-old APP_swe_/PS1 transgenic male mice and, as controls, to age-matched C57BL/6J mice was beneficial under several respects with no concurrent microglial activation in both earlier and later phases of the neuropathology in AD-model mice [280].

Brain pericytes are cells enclosed within the basement membranes of capillaries that help regulate blood flow, blood-brain barrier (BBB) permeability, and angiogenesis. Under basal (untreated) conditions pericytes express the mRNAs of a complex set of PRRs, including NOD1, NOD2, NLRC5, NLRP1-3, NLRP5, NLRP9, NLRP10/PYNOD, and NLRX. Exposure to PAMPs or DAMPs increased the levels of NOD2 and elicited the expression of NLRA and NLRC4 too. TLR-2 and TLR-9 were also affected as well as inflammasome-forming caspases and inflammasome-cleaved interleukins. Therefore, it is likely that pericytes play a relevant controlling role in neuroinflammation [218].

Human brain endothelial cells also express a complex set of PRRs, including NOD1, NOD2, NLRC4, NLRC5, NLRP1, NLRP3, NLRP5, NLRP9, NLRP10/PYNOD, NLRP12, NLRA, and NLRX, whose expression can be significantly affected by PAMPs or DAMPs, leading to the activation of inflammasomes and the subsequent caspase-1-cleaved interleukins IL-1β and IL-33 maturation. Therefore, cerebral endothelial cells might crucially partake in neuroimmune and neuroinflammatory processes at the BBB level [219].

#### 3.3.8. NLRP10/PYNOD Inflammasome

Following either in-vitro or in-vivo Aβs exposure, the levels of NLRP10/PYNOD, another NOD-like protein, declined significantly being broken down by the activated cathepsins. Adding recombinant NLRP10/PYNOD to Aβ-exposed glial cultures scaled down caspase 1 activation and IL-1β release indicating that cytosolic cathepsin cleavage of NLRP10 is a prerequisite for the formation of NLRP3 inflammasome [264].

In sharp contrast, in an acute ischemic stroke/reperfusion model, NLRP10/PYNOD was overexpressed in the penumbra zone of wild-type animals, whereas in NLRP10/PYNOD knockout mice after ischemic stroke/reperfusion the neuronal apoptosis, the activation of glial cells, and the proinflammatory agents’ levels were reduced, and the TLR-4/NF-κB signaling pathways markedly suppressed in the hippocampus. Moreover, in cultured LPS-treated NLRP10/PYNOD knockout mouse astrocytes the NLRP12/ASC/Caspase-1 and TLR-4/NF-κB pathways were hampered being rescued by an overexpressed NLRP10/PYNOD [281].

A recent study from Zeng et al. [282] revealed the antinflammatory and antineurotoxic effects of NLRP10/PYNOD, a NOD (NLR)-like receptor protein, in the LPS-induced murine microglial BV-2 cells transfected with pEGFP-C2-PYNOD. Overexpressed NLRP10/PYNOD dose-dependently (i) hampered NOS-2 protein induction and thus prevented the cytotoxic effects of NO overrelease; (ii) cut down caspase-1 activation and hence IL-1β synthesis and secretion; (iii) blocked the nuclear translocation of NF-κB p65; and (iv) mitigated the growth-hindering and apoptosis-advancing effects of BV-2 cells on SK-N-SH cells. Whether PYNOD inflammasome has any role in human AD-related neuroinflammation will be determined by further studies.

### 3.4. C-Type Lectin (Dectin-1, CLEC-2) Receptors (CLRs)

CLRs have diverse functions ranging from embryonic development to immune function. One subgroup of CLRs is the Dendritic cell (DC)-associated C-type lectin-1 (Dectin-1) cluster, comprising of seven receptors including MICL, CLEC-2, CLEC-12B, CLEC-9A, MelLec, Dectin-1 and LOX-1.

The Dectin-1 cluster of receptors has a broad range of ligands and functions. Dectin-1 is an immune-receptor tyrosine-based activation motif (ITAM)-coupled CLR. It senses several DAMPs, recruits the spleen tyrosine kinase (Syk) and lets out proinflammatory signals [283]. Dectin-1 activated the NLRP3 inflammasome for anti-fungal defense and increased IL-1β synthesis via a non-canonical caspase-8 inflammasome [208,284]. After ischemic stroke or spinal cord injury an upregulated Dectin-1 drove through the Syk signaling pathway activation microglia, neuroinflammation, demyelination, and axons damage [285,286]. Conversely, the Dectin-1/Syk pathway inhibition mitigated microglial activation, the brain infarct volume, neurological damage, proinflammatory cytokines production, TNF-α, and NOS-2 expression. Conversely, β-glucan/Dectin-1 signaling induced a beneficial neuroinflammation promoting the regeneration of traumatically severed retinal ganglion cell axons in mice [287]. Dectin-1 is upregulated in human AD tissues [288,289] and AD mouse models [290,291]. In the amyloidogenic 5XFAD transgenic mouse model Dectin1 helped preserve microglia in a homeostatic state because it can induce intracellular signals like those of TREM2 [95].

Recent data have led some authors to propose that changes in the gut microbiota and an increased intestinal permeability might be instrumental in AD pathogenesis. Zonulin is a key modulator that regulates intestinal barrier function. Moreover, the activation of C-type lectin-like immune receptor 2 (CLEC-2), a platelet surface receptor may occur in AD. Significant increases in CLEC-2 and Zonulin levels, as assessed via ELISA assays, occurred in 110 MCI and 110 AD patients vs. 110 controls with no dementia. Furthermore, MCI patients had lower CLEC-2 and Zonulin levels than frank AD patients. Further studies will clarify the potential import of these findings in AD [292].

### 3.5. RIG-I (Retinoid Acid-Inducible Gene-I)-Like Receptors

Human and murine neurons, astrocytes, and microglia constitutively express the retinoic acid-inducible gene I (RIG-I), which is a key cytosolic immune PRR. As a sensor, RIG-I detects 5’-PPP double-stranded RNAs produced by a variety of viruses and intracellular bacteria. Therefore, RIG-I is responsible for mounting a response against infectious agents besides partaking in neuroinflammatory processes linked with neurodegenerative diseases [293,294].

RIG-I expression becomes more intense in the course of a bacterial or viral infection in a pathogen and cell type-specific manner. For example, both surface and cytosolic PRR ligands, like bacterial and viral RNA and DNA, effectively increase RIG-I expression, RIG-I-dependent Interferon Regulatory Factor 3 (IRF3) phosphorylation, and subsequent type I Interferon (IFN) production in human microglia [295,296]. Studies on the recognition of Japanese encephalitis virus (JEV) RNA have revealed that via RIG-I neurons are one of the sources of several proinflammatory agents, such as IL-6, IL-12, p70, MCP-1, IP-10, and TNF-α, in JEV-infected brain. Conversely, animals with RIG-I knockout neurons had an increased viral load and released lesser amounts of cytokines/chemokines [297]. Interestingly, CoCl_2_-induced chemical hypoxia increased RIG-I expression and the production of proinflammatory IL-1β, IL-6, and TNF-α via the interaction of Interferon-Β Promoter Stimulator-1 (IPS-1) and TNF receptor-associated factor 6 (TRAF6) and the NF-κB pathway in in-vitro human astrocytes [298]. Finally, a study exploring innate immune proteins expression levels in human temporal and occipital cortices proved that RIG-1 expression is significantly heightened in the temporal cortex and plasma of patients with mild cognitive impairment (MCI). Moreover, an exposure to the RIG-1 ligand 5’ppp RNA increased the expression of APP and Aβs in primary human astrocytes. These results have revealed a potential implication of RIG-1 in the MCI phase of AD development [294].

### 3.6. Calcium-Sensing Receptor (CaSR)

A component of Family C G-protein-coupled receptors (GPCRs), the CaSR is a ubiquitously expressed cationic multiligand receptor and at the same time a DAMP-sensing receptor implicated in several inflammatory diseases [74,248,299]. The first cloning of the highly conserved *CaSR* gene was from rat parotid glands. CaSR acts as a calciostat rapidly sensing changes in systemic extracellular Ca^2+^ levels ([Ca^2+^]_e_) [300]. It is located at the plasmalemma and can signal both thence and, after endocytosis, intracellularly [301]. Besides Ca^2+^, its hugely bilobed extracellular domain, named *Venus flytrap*, binds several other agonists such as mono-, di-, and trivalent ions; amino acids (e.g., phenylalanine; tryptophan); and polycationic agonists such as polyamines; aminoglycoside antibiotics; and last but not least Aβs pathological aggregates [13,66,299] (Figure 2). A seven-pass transmembrane domain (the 7TM) joins the *Venus flytrap* to the intracellular domain (ICD). Ligand binding at the CaSR’s *Venus flytrap* engenders a signal that crosses the 7TM domain to reach the ICD and induce its binding to G-proteins such heterotrimeric G_i/o_, G_q/11_, G_12/13_, and G_s_, and low molecular weight Arf6, RhoA, Ras, Rab1 and Rab11a. The intervening 7TM domain plays a critical role in the activation or suppression of agonist-evoked CaSR signaling as it holds specific binding pockets for CaSR positive allosteric modulators (PAMs or *calcimimetics*) and for CaSR negative allosteric modulators (NAMs or *calcilytics*) [302,303,304]. The ligand CaSR/G-protein mechanisms drive an intricate set of signaling pathways, conditioned by the nature of the ligand, the cell type considered, and the specific G-protein involved. Such intracellular signals make use of (i) transcription factors (TFs); (ii) protein kinases (AKT, PKCs, MAPKs); (iii); phospholipases (A_2_, C, and D); (iv) second messenger up- or downregulation (e.g., cAMP); and (v) Ca^2+^ influxes via TRPC6-encoded receptor-operated ion channels [305,306,307]. While functioning as a calciostat the CaSR controls systemic Ca^2+^ homeostasis via the modulation of parathyroid hormone (PTH) and active Vitamin D3 release, which regulate intestinal Ca^2+^ absorption, skeletal Ca^2+^ storage, and kidney Ca^2+^ resorption [306,307].

Several earlier works reported that tissue inflammation can activate CaSR signaling and, on the other hand, that CaSR signaling can activate tissue inflammation. This occurs in deep skin burn wounds [308]; allergen-sensitized airways of mice and of human asthmatic patients [309]; hypertension-induced rat aorta dysfunctional remodeling [310]; LPS-treated rodent lungs [311]; rheumatoid arthritis monocytes [312]; and a variety of diseases of the adipose tissue [313]; kidneys [314]; colon-rectum [315]; and prostate [316]. Of note, CaSR NAM NPS 2143 elicited an effective anti-inflammatory action in asthma [309] and in LPS-evoked pneumonia [311].

Local gradients of [Ca^2+^]_e_ increases due to cation releases from cells undergoing necrosis following injury or infection activate CaSR’s signaling on mature monocytes and/or macrophages. This attracts the phagocytic cells toward the high [Ca^2+^]_e_ injury sites, a process the chemokine MCP-1/CCR2 signaling aids by increasing the cells’ CaSR expression in a series of positive feed-back cycles [317]. Notably, IL-1β and IL-6 too upregulate CaSR expression in parathyroid glands, kidneys, and human astrocytes by acting upon specific response elements of the *CASR* gene promoters [318]. Besides, CaSR’s activation drives Ca^2+^ release from the ER [319].

Therefore, a heightened [Ca^2+^]_e_ works as a DAMP that activates the NLRP3 inflammasome via G protein coupled CaSR signaling [320] and via the phosphatidyl inositol/Ca^2+^ pathway in association with a concurrent fall in cAMP levels in monocytes and macrophages. Thus, CaSR signaling does partake in the induction of inflammation in human cryopyrin-associated periodic syndromes (CAPs) and in mouse models of carrageenan-evoked swelling of foot pads [74,248]. In monocytes of rheumatoid arthritis and of hypertension-evoked aortic remodeling patients [310] [Ca^2+^]_e_ increases also trigger the NLRP3 inflammasome signaling via CaSR activation [312] (Figure 2). Moreover, in macrophages and monocytes [Ca^2+^]_e_ increases also activate the macropinocytosis process and the uptake of MDP (a NOD2 ligand) via CaSR-mediated Gα-protein signaling—both events being followed by phosphorylation of the NF-kB p65 subunit or otherwise blocked by the CaSR NAM NPS 2143 or by removing the extracellular Ca^2+^ [312,321].

As in the other organs, CaSR’s expression is ubiquitous in the brain [322], being most intense in hippocampal neurons, astrocytes, microglia, and ependymal cells [323]. Moreover, in the CNS the CaSR crucially modulates nerve cells mitotic activity, prenatal migration, and differentiation; postnatal neurotransmitters release from synapses [322,323,324,325,326]; K^+^ fluxes [327,328]; and L-amino acid sensing [329].

Importantly, CaSR’s expression increases and its function evokes harmful effects in conditions of *acute* CNS damage entailing a subsequent neuroinflammation, such as ischemia/hypoxia/stroke and subarachnoid hemorrhage (which also causes an ischemia/hypoxia through local tissue compression). In adult Kunming mice subjected to a 2-h-long focal cerebral ischemia via carotid ligation followed by a 22-h-long reperfusion, the activation of the JNK/P38 MAPK signaling pathway increased CaSR expression and neurons’ apoptosis, both of which were further boosted by administering Gadolinium trichloride (GdCl_3_), a CaSR PAM [330]. In a mouse model of subarachnoid hemorrhage, GdCl_3_ worsened the brain edema, the extent of neurodegeneration, and the intensity of neurological deficits, whereas the administration of NPS 2143, a CaSR NAM, remarkably reduced all such injuries and deficits. Moreover, NPS 2143 countered the GdCl_3_-evoked increases in NLRP3 inflammasome signaling, caspase-1 activity, IL-1β synthesis, and CaMKII activity and rescued adenylyl cyclase activity and cAMP normal levels. Of note, KN-93, a selective inhibitor of CaMKII, acted by itself as protectively as CaSR NAM NPS 2143 did, indicating its connection with CaSR signaling [331]. Furthermore, traumatic brain injuries too entailed a CaSR’s heightened expression and signaling activity that hampered GABA_B_R‘s inhibitory signaling and hence intensified a noxious hyper excitatory activity of the neurons. Mimicking the protective effects of hypothermia, giving CaSR NAM NPS 89,636 did remarkably reduce trauma-evoked brain tissue damage and motor function disability [332]. However, the latter authors did not assess inflammasome expression in their model. In all the acute conditions just mentioned, hypoxia raised [Ca_2_^+^]_i_ and boosted CaSR expression thereby increasing BACE1/β-secretase activity, which resulted in the toxic overproduction of Aβ_42_s. Again, intraventricularly administered CaSR NAM Calhex 231 mitigated such detrimental effects of hypoxia [333].

Studies employing preclinical models of AD in vitro (“*in a Petri dish*”) made of either NAHAs or postnatal HCN-1A neurons isolated from human cerebral cortex fragments showed that exogenous fAβs or sAβs form Aβ•CaSR complexes that are rapidly endocytosed [11,14,33,301]. The thus induced Aβ•CaSR signaling drove (i) a transient CaSR overexpression in vitro [33], which however escalated with time in the hippocampal neurons and astrocytes of 3xTg [334] and of B6C3-Tg (APP_swe_/PSEN1dE9) AD-model mice [335]; (ii) a decreased proteasome’s function promoting the intracellular accumulation of toxic Aβs in human neurons and astrocytes [33]; (iii) a shift of human APP’s metabolism along the amyloidogenic processing (AP) leading to the synthesis and release of neurotoxic Aβ_42_-os surpluses which deeply downregulated the extracellular shedding of neurotrophic and neuroprotective soluble (s)APP-α [67]; (iv) the hyperphosphorylation of Tau proteins (p-Taues) by raising the glycogen synthase kinase (GSK)-3β activity [302]; (v) the induction and activation of NOS-2 producing toxic NO surpluses [336]; (vi) the overproduction and instant release of vascular endothelial growth factor (VEGF)-A_169_ from NAHAs, which would cause BBB’s dysfunction in vivo [337]; (vii) an upregulated NAHAs’ synthesis and release/shedding of proinflammatory IL-6 (Figure 2); InterCellular Adhesion Molecule-1 (ICAM-1; both its holoprotein and soluble fragment); Regulated upon Activation, normal T cell Expressed and presumably Secreted (RANTES); and Monocyte Chemotactic Protein (MCP)-2. Importantly, CaSR NAM NPS 2143 totally suppressed the Aβ•CaSR-elicited oversecretion of IL-6 and partially yet significantly that of ICAM-1, RANTES, and MCP-2, proving that human astrocytes Aβ•CaSR signaling could directly advance AD’s neuroinflammation [13]; (viii) the slow yet progressive death of the human cortical neurons, which was fully prevented by CaSR NAM NPS 2143; conversely, the NAHAs survived and kept producing and releasing all the above-mentioned detrimental factors [33]. It is also worth mentioning here that the CaSR PAM (*calcimimetic*) NPS R-568 administration intensified the release of Aβ_42_-os from the NAHAs further confirming the noxious role of Aβ•CaSR signaling in AD amyloidosis [33].

Interestingly, Feng et al. [335] reported that CaSR NAM NPS 2143 prevented the loss of neuronal dendritic filopodia and synaptic spines, the downregulation of the presynaptic marker synaptotagmine-1, and of the postsynaptic marker PDS 95, and mitigated cognitive deficits induced by Aβ_1-42_-os in B6C3-Tg (APP_swe_/PSEN1dE9) AD-model mice and in cultured primary hippocampal neurons.

## 4. Concluding Remarks

Abundant evidences show that a trio of mutually interacting pathogenetic factors, i.e., oligomers and polymers of Aβ and p-Tau, and a concurrent chronic neuroinflammation drive the progressive development and spreading of human AD. Certainly, the several families of Aβ-binding danger/damage sensing receptors considered here (and other receptors reviewed elsewhere; see [12,299] bear witness to the quite high complexity of the mechanisms underlying the development and progression of AD neuropathology. The intricacy of the picture is further heightened by the different molecular aggregations Aβs and p-Taues can take, by key genetic mutations in early-onset/familial AD, by background genetic predispositions in late-onset/sporadic AD, by age-related metabolic and vascular diseases, and by striking morpho-functional differences, particularly at the cerebral cortical and subcortical levels, distinguishing human neural cells and their connectome from their animal counterparts. Moreover, the signaling pathways activated by the various plasma membrane and intracellular danger-sensing/binding receptors might differ among living species and may mutually crosstalk in several ways, resulting in dissimilar upshots. In addition, differences in cell types and models used for experimental studies may elicit divergent outcomes and hence conclusions.

As the human brain is the most complex living structure in existence, it is no wonder that the molecular mechanisms underlying its ailments feature extreme levels of intricacy and that the so-far tested therapeutic agents have met with failures. Clinical trials attempting to modify PRRs activities in AD patients are under course in relation to RAGE, TREM2, and NLRP3, whereas a trial about CD36 gave inconclusive results for lack of evidence (Table 4).

Not surprisingly, the recently increased availability of funds has induced a surge in the number of novel pathogenetic theories about AD (Table 1). On the other hand, the innate immunity has a long evolutionary history culminating in mammals, as through the PRRs it safeguards against harm from quite high numbers of PAMPs and/or DAMPs. The key question one should ponder is whether there is any so far hidden (to our eyes) hierarchical system according to predetermined rankings that might regulate the apparent mess of PRRs and signaling mechanisms thereof that is crucial for AD onset and progression. This question inevitably leads to the old controversy about which is the first driver or *primum movens* of late-onset AD. The evidences gained from the experimental use of preclinical in-vitro (”in a Petri dish”) AD-models made of nontumorigenic human neurons and astrocytes indicate that the first AD drivers are the soluble or fibrillar Aβs, which, however, act alone for a very short time, as they directly induce the production and release of additional amounts of Aβs and concurrently of the other two main AD drivers, i.e., p-Taues and neuroinflammation [13,33,299,302]. In the same preclinical in-vitro AD-models a trio of proinflammatory cytokines—i.e., IL-1β, TNF-α, and IFN-γ—did not increase the rate of de novo Aβs synthesis but only expedited their release from the human neural cells [33]. Moreover, AD is a progressively intra-brain spreading disease and the mechanism through which it propagates must also be crucial (Figure 1 and Figure 2). To achieve all this, it would suffice that Aβs interacted with some topmost ranking or hierarchically supreme PRR. The potential candidates might belong to any of the main PRR families previously considered. However, the hypothesis that a Ca^2+^ dyshomeostasis promotes AD development has been circulating for years [342]. We believe that there is truth in it, though one should see it from a slightly shifted angle while taking into account the multiligand (Aβs included) and multisignaling CaSR. Based on the highly beneficial effects of specific NAMs of the CaSR given to soluble or fibrillar Aβ-exposed nontumorigenic human cortical neurons and astrocytes in culture, it seems likely that the CaSR is endowed with all the bona fide features proper of a hierarchically very highly placed if not supreme Aβs-binding PRR. However, it cannot be neglected that some coreceptor heterodimerizing with the CaSR or crosstalk of the CaSR with other PRRs might also play noteworthy roles in the development and progression of LOAD/SAD. Instead, in the early-onset/familial AD cases the mutated gene responsible for the Aβ overproduction is the uncontested *primum movens.* Yet, even in this hereditary AD form the CaSR interaction with the overproduced Aβs is likely to significantly aggravate the brain amyloid burden by further intensifying the overproduction of Aβs and of all the other noxious agents its signaling induces, thus detrimentally accelerating the clinical course. Future studies and clinical trials will test the validity of this Aβs•CaSR signalling-based hypothesis and the entailed therapeutic remedies for hitherto unforgiving Alzheimer’s disease.

## Figures and Tables

**Table 1:** Time-line hypotheses regarding the causes of SAD/LOAD.

Year *		Refs.
1976	Cholinergic hypothesis	[20]
1991	Amyloid-β hypothesis	[21,22]
1992	Calcium dyshomeostasis hypothesis	[23]
1992	Inflammation hypothesis	[24]
1994	Metal ions hypothesis	[25]
1997	Aβ•CaSR activating Ca^2+^ channels hypothesis	[26]
2000–2004	Neurovascular hypothesis	[27,28,29]
2004	Mitochondrial hypothesis	[30]
2004	Glymphatic system hypothesis	[31]
2009	Tau propagation hypothesis	[32]
2013–2020	Aβ•CaSR driving AD progression hypothesis	[33]
2018	Cellular senescence	[34]
2020	Neuroimmunomodulation hypothesis	[35]

* The year refers to the first offering of the hypothesis; SAD, sporadic Alzheimer’s disease; LOAD, late-onset Alzheimer’s disease; CaSR, calcium sensing receptor.

**Table 2:** Main factors increasing the risk of SAD/LOAD.

Family history	[36]
Apolipoprotein-ε4 genotype	[37]
Metabolic syndrome Midlife obesity Hypercholesterolemia Hyperhomocysteinemia Type 2 Diabetes	[38,39,40,41,42]
Oxidative stress	[42]
Midlife hypertension	[43]
Sleep disorders	[44]
Oral infections	[45]
Gut microbiome dysbiosis	[46]
Human immunodeficiency virus (HIV)	[47]
Herpes simplex virus type 1 (HSV-1)	[48]

**Table 3:** The main inflammasomes expressed in human CNS cell types, in human AD brains and in AD-model animal brains.

	NLRP1	NLRP2	NLRP3	NAIP/ NLRC4	AIM2	IPAF	NOD1	NOD2	NLRP10/ PYNOD	Refs. ^#^
**Neurons**	+ ‡		+		+					[212,215,217]
**Astrocytes**		+	+	+		+				[210,212,216]
**Microglia**	+		+							[212]
**Oligo-dendrocytes**	+		+							[214]
**Pericytes**	+	+	+	+			+	+		[218]
**Endothelial cells**	+		+	+			+	+		[219]
**Postmortem** **AD human brain**	+		+	+		+				[215,226,256,272,274,275]
**AD animal models**	+	+	+	+	+				+	[227,228,229, 249,250,255,257,258,259,260,261,262,267,273,278]

^#^ Refs., reference numbers. + ^‡^, reported inflammasome in relation to AD neuroinflammation.

**Table 4:** Clinical trials targeting PPRs in the treatment of AD.

Target	Treatment	U.S. FDA Status:	Study Status	Refs.
RAGE	*Azeliragon/PF-04494700* (oral RAGE inhibitor)	AD (Phase 2/3)	Active	[338]
TREM2	*AL002* (anti-human TREM2)	AD (Phase 1/2)	Active	[339]
NLRP3	*Inzomelid* (oral, brain-penetrant inhibitor of inflammasomes containing NLRP3)	AD (Phase 1)	Completed in March 2020	[340]
CD36	*Pioglitazone* (thiazolidinedione peroxisome-proliferator activated receptor γ [PPARγ] agonist)	Mild Cognitive Impairment (Phase 3)	Terminated (Lack of efficacy of the drug)	[341]

## Abbreviations

Aβs	Amyloid-β peptides
Ab(RAGE)	anti-RAGE antibody
AD	Alzheimer’s disease
AGEs	advanced glycation endproducts
AIM2	absent in melanoma 2 protein
APOE	Apolipoprotein E
APP	amyloid precursor protein
ASC	apoptosis-associated speck-like adaptor protein containing a CARD
Aβ-os	Aβ oligomers
BBB	blood-brain barrier
BECs/CECs	brain/cerebral endothelial cells
C1q	complement component 1q
CAA	cerebral amyloid angiopathy
cAMP	3′,5′-cyclic adenosine monophosphate
*CARD*	caspase activation and recruitment *domain*
CaSR	calcium-sensing receptor
CCL	C-C Motif Chemokine Ligand
CLEC-2	C-type lectin-like immune receptor 2
CLRs	C-type lectin receptors
CNS	central nervous system
CSF	cerebrospinal fluid
DAMPs	damage-associated molecular patterns
DAP12/TYROBP	DNAX activating protein
Dectin-1	Dendritic cell (DC)-associated C-type lectin-1 cluster
DS	Down’s syndrome
ERK1/2	extracellular signal-regulated kinase 1/2
GdCl_3_	Gadolinium trichloride
GFAP	glial fibrillar acidic protein
GMF	glia maturation factor
GPCRs	G-protein-coupled receptors
GSK-3β	glycogen synthase kinase-3β
HDL	high-density lipoprotein
HMGB1	high-mobility group box B1
hNSCs	human neural stem cells
ICAM-1	Intercellular Adhesion Molecule-1
IPAF	ICE protease-activating factor
ITAM	immune-receptor tyrosine-based activation motif
JEV	Japanese encephalitis virus
JNK	c-JUN terminal kinase
LPS	lipopolysaccharide
MAPK	mitogen activated protein kinase
MCI	mild cognitive impairment
MCP	Monocyte Chemotactic Protein
MDP	muramyl dipeptide
mTOR	mammalian target of rapamycin
NACHT	NAIP plus CIITA (MHC class II transcription activator) plus HET-E (incompatibility locus protein from *Podospora anserina*) and TP1 (telomerase-associated protein)
NAHAs	nontumorigenic adult human astrocytes
NAIP	neuronal apoptosis inhibitor protein
NAM	negative allosteric modulator
NF-κB	nuclear factor-κB
NFT	neurofibrillary tangle
NLR	NOD-like receptor
NLRP	Nucleotide-binding domain, leucine-rich repeat and PYRIN domain containing protein
NO	nitric oxide
NOD	nucleotide-binding oligomerization domain
NOS-2	nitric oxide synthase-2
oxLDLs	oxidized low-density lipoproteins
p-Tau-es	hyperphosphorylated Tau proteins
p-Tau-os	p-Tau oligomers
PAMPs	pathogen-associated molecular patterns
PAMs	positive allosteric modulators
Panx-1	Pannexin 1 protein
PI3K	Phosphoinositide 3-kinase
PICs	proinflammatory cytokines
PKR	double-stranded RNA-activated protein kinase
PPRs	pattern recognition receptors
PYHIN	N-terminal pyrin domain (PYD) plus hemopoietic inducible nuclear (HIN) proteins
PYRIN/DAPIN	Domain in Apoptosis and Interferon response
RAGE	receptor for AGEs
RANTES	Regulated upon Activation, normal T cell Expressed and presumably Secreted
RCT	reverse cholesterol transport
RIG	retinoic acid-inducible gene
ROS	reactive oxygen species
SRs	scavenger receptors
sTREM2	soluble TREM2
Syk	spleen tyrosine kinase
TAM	Tyro3, Axl, and Mer receptor
TLRs	Toll-like receptors
Toll-like	similar to *Drosophila Toll* gene
TREM	triggering receptor expressed on myeloid cells
VEGF-A	vascular endothelial growth factor-A

## References

[1] Prince M.J., Wimo A., Guerchet M.M., Ali G.C., Wu Y.-T., Prina M. World Alzheimer Report 2015. The Global Impact of Dementia: An Analysis of Prevalence, Incidence, Cost and Trends. https://www.alzint.org.

[2] Knopman D.S., Haeberlein S.B., Carrillo M.C., Hendrix J.A., Kerchner G., Margolin R., Maruff P., Miller D.S., Tong G., Tome M.B. (2018). The National Institute on Aging and the Alzheimer’s Association Research framework for Alzheimer’s disease: Perspectives from the research roundtable. Alzheimers Dement..

[3] Labzin L.I., Heneka M.T., Latz E. (2018). Innate immunity and neurodegeneration. Annu. Rev. Med..

[4] Becher B., Spath S., Goverman J. (2017). Cytokine networks in neuroinflammation. Nat. Rev. Immunol..

[5] Gouras G.K., Olsson T.T., Hansson O. (2015). β-Amyloid pep–tides and amyloid plaques in Alzheimer’s disease. Neurotherapeutics.

[6] Bloom G.S. (2014). Amyloid-β and tau: The trigger and bullet in Alzheimer disease pathogenesis. JAMA Neurol..

[7] Della Bianca V., Dusi S., Bianchini E., Dal Pra I., Rossi F. (1999). Beta-amyloid activates the O^−2^ forming NADPH oxidase in microglia, monocytes, and neutrophils: A possible inflammatory mechanism of neuronal damage in Alzheimer’s disease. J. Biol Chem..

[8] Butterfield D.A., Boyd-Kimball D. (2018). Oxidative stress, amyloid-β peptide, and altered key molecular pathways in the pathogenesis and progression of Alzheimer’s Disease. J. Alzheimers Dis..

[9] Chiarini A., Dal Pra I., Menapace L., Pacchiana R., Whitfield J.F., Armato U. (2005). Soluble amyloid β-peptide and myelin basic protein strongly stimulate, alone and in synergism with joint proinflammatory cytokines, the expression of functional nitric oxide synthase-2 in normal adult human astrocytes. Int. J. Mol. Med..

[10] Dal Prà I., Armato U., Chioffi F., Pacchiana R., Whitfield J.F., Chakravarthy B., Gui L., Chiarini A. (2014). The Aβ peptides-activated calcium-sensing receptor stimulates the production and secretion of vascular endothelial growth factor-A by normoxic adult human cortical astrocytes. Neuromol. Med..

[11] Dal Prà I., Chiarini A., Pacchiana R., Gardenal E., Chakravarthy B., Whitfield J.F., Armato U. (2014). Calcium-Sensing Receptors of Human Astrocyte-Neuron Teams: Amyloid-β-Driven Mediators and Therapeutic Targets of Alzheimer’s Disease. Curr. Neuropharmacol..

[12] Chiarini A., Armato U., Liu D., Dal Prà I. (2016). Calcium-sensing receptors of human neural cells play crucial roles in Alzheimer’s disease. Front. Physiol..

[13] Chiarini A., Armato U., Hu P., Dal Prà I. (2020). CaSR Antagonist (Calcilytic) NPS 2143 Hinders the Release of Neuroinflammatory IL-6, Soluble ICAM-1, RANTES, and MCP-2 from Aβ-Exposed Human Cortical Astrocytes. Cells.

[14] Dal Prà I., Armato U., Chiarini A. Specific interactions of calcium-sensing receptors (CaSRs) with soluble amyloid-β peptides—A study using cultured normofunctioning adult human astrocytes. Proceedings of the 2nd International Symposium on the Calcium-sensing Receptor.

[15] Dal Prà I., Chiarini A., Gui L., Chakravarthy B., Pacchiana R., Gardenal E., Armato U. (2015). Do astrocytes collaborate with neurons in spreading the “infectious” Aβ and Tau drivers of Alzheimer’s disease?. Neuroscientist.

[16] Calsolaro V., Edison P. (2016). Neuroinflammation in Alzheimer’s disease: Current evidence and future directions. Alzheimers Dement..

[17] Hardy J., Selkoe D.J. (2002). The amyloid hypothesis of Alzheimer’s disease: Progress and problems on the road to therapeutics. Science.

[18] Braak H., Del Tredici K. (2015). The preclinical phase of the pathological process underlying sporadic Alzheimer’s disease. Brain.

[19] Calhoun A., King C., Khoury R., Grossberg G.T. (2018). An evaluation of memantine ER + donepezil for the treatment of Alzheimer’s disease. Expert Opin. Pharmacother..

[20] Davies P., Maloney A.J. (1976). Selective loss of central cholinergic neurons in Alzheimer’s disease. Lancet.

[21] Selkoe D.J. (1991). The molecular pathology of Alzheimer’s disease. Neuron.

[22] Hardy J., Allsop D. (1991). Amyloid deposition as the central event in the aetiology of Alzheimer’s disease. Trends Pharmacol. Sci..

[23] Mattson M.P., Cheng B., Davis D., Bryant K., Lieberburg I., Rydel R.E. (1992). beta-Amyloid peptides destabilize calcium homeostasis and render human cortical neurons vulnerable to excitotoxicity. J. Neurosci..

[24] McGeer P.L., Rogers J. (1992). Anti-inflammatory agents as a therapeutic approach to Alzheimer’s disease. Neurology.

[25] Bush A.I., Pettingell W.H., Multhaup G., d Paradis M., Vonsattel J.P., Gusella J.F., Beyreuther K., Masters C.L., Tanzi R.E. (1994). Rapid induction of Alzheimer A beta amyloid formation by zinc. Science.

[26] Ye C., Ho-Pao C.L., Kanazirska M., Quinn S., Rogers K., Seidman C.E., Seidman J.G., Brown E.M., Vassilev P.M. (1997). Amyloid-beta proteins activate Ca(2+)-permeable channels through calcium-sensing receptors. J. Neurosci. Res..

[27] Kalaria R.N. (2000). The role of cerebral ischemia in Alzheimer’s disease. Neurobiol. Aging.

[28] De la Torre J.C. (2002). Alzheimer disease as a vascular disorder: Nosological evidence. Stroke.

[29] Iadecola C. (2004). Neurovascular regulation in the normal brain and in Alzheimer’s disease. Nat. Rev. Neurosci..

[30] Swerdlow R.H., Khan S.M. (2004). A “mitochondrial cascade hypothesis” for sporadic Alzheimer’s disease. Med. Hypotheses.

[31] Deane R., Wu Z., Sagare A., Davis J., Du Yan S., Hamm K., Xu F., Parisi M., LaRue B., Hu H.W. (2004). LRP/amyloid beta-peptide interaction mediates differential brain efflux of Abeta isoforms. Neuron.

[32] Frost B., Jacks R.L., Diamond M.I. (2009). Propagation of tau misfolding from the outside to the inside of a cell. J. Biol. Chem..

[33] Armato U., Chiarini A., Chakravarthy B., Chioffi F., Pacchiana R., Colarusso E., Whitfield J.F., Dal Prà I. (2013). Calcium-sensing receptor antagonist (calcilytic) NPS 2143 specifically blocks the increased secretion of endogenous Aβ42 prompted by exogenous fibrillary or soluble Aβ25-35 in human cortical astrocytes and neurons—Therapeutic relevance to Alzheimer’s disease. Biochim. Biophys. Acta.

[34] Baker D.J., Petersen R.C. (2018). Cellular senescence in brain aging and neurodegenerative diseases: Evidence and perspectives. J. Clin. Investig..

[35] Maccioni R.B., Navarrete L.P., González A., González-Canacer A., Guzmán-Martínez L., Cortés N. (2020). Inflammation: A major target for compounds to control Alzheimer’s Disease. J. Alzheimers Dis..

[36] Silverman J.M., Raiford K., Edland S., Fillenbaum G., Morris J.C., Clark C.M., Kukull W., Heyman A. (1994). The consortium to establish a registry for Alzheimer’s disease (CERAD). Part VI. Family history assessment: A multicenter study of first-degree relatives of Alzheimer’s disease probands and nondemented spouse controls. Neurology.

[37] Liu C.C., Liu C.C., Kanekiyo T., Xu H., Bu G. (2013). Apolipoprotein E and Alzheimer disease: Risk, mechanisms and therapy. Nat. Rev. Neurol..

[38] Razay G., Vreugdenhil A., Wilcock G. (2007). The Metabolic Syndrome and Alzheimer Disease. Arch. Neurol..

[39] Dias H.K., Brown C.L., Polidori M.C., Lip G.Y., Griffiths H.R. (2015). LDL-lipids from patients with hypercholesterolaemia and Alzheimer’s disease are inflammatory to microvascular endothelial cells: Mitigation by statin intervention. Clin. Sci..

[40] Whitmer R.A., Gunderson E.P., Barrett-Connor E., Quesenberry C.P., Yaffe K. (2005). Obesity in middle age and future risk of dementia: A 27 year longitudinal population based study. BMJ.

[41] Zhuo J.M., Wang H., Praticò D. (2011). Is hyperhomocysteinemia an Alzheimer’s disease (AD) risk factor, an AD marker, or neither?. Trends Pharmacol. Sci..

[42] Butterfield D.A., Halliwell B. (2019). Oxidative stress, dysfunctional glucose metabolism and Alzheimer disease. Nat. Rev. Neurosci..

[43] Launer L.J., Ross G.W., Petrovitch H., Masaki K., Foley D., White L.R., Havlik R.J. (2000). Midlife blood pressure and dementia: The Honolulu-Asia aging study. Neurobiol. Aging.

[44] Osorio R.S., Pirraglia E., Agüera-Ortiz L.F., During E.H., Sacks H., Ayappa I., Walsleben J., Mooney A., Hussain A., Glodzik L. (2011). Greater risk of Alzheimer’s disease in older adults with insomnia. J. Am. Geriatr. Soc..

[45] Olsen I., Singhrao S.K. (2015). Can oral infection be a risk factor for Alzheimer’s disease?. J. Oral Mcrobiol..

[46] Vogt N.M., Kerby R.L., Dill-McFarland K.A., Harding S.J., Merluzzi A.P., Johnson S.C., Carlsson C.M., Asthana S., Zetterberg H., Blennow K. (2017). Gut microbiome alterations in Alzheimer’s disease. Sci. Rep..

[47] Canet G., Dias C., Gabelle A., Simonin Y., Gosselet F., Marchi N., Makinson A., Tuaillon E., Van de Perre P., Givalois L. (2018). HIV Neuroinfection and Alzheimer’s Disease: Similarities and Potential Links?. Front. Cell. Neurosci..

[48] Lövheim H., Gilthorpe J., Adolfsson R., Nilsson L.G., Elgh F. (2015). Reactivated herpes simplex infection increases the risk of Alzheimer’s disease. Alzheimers Demen..

[49] Saez-Atienzar S., Masliah E. (2020). Cellular senescence and Alzheimer disease: The egg and the chicken scenario. Nat. Rev. Neurosci..

[50] Han X., Zhang T., Liu H., Mi Y., Gou X. (2020). Astrocyte senescence and Alzheimer’s Disease: A review. Front. Aging Neurosci..

[51] Yiannopoulou K.G., Papageorgiou S.G. (2020). Current and Future Treatments in Alzheimer Disease: An Update. J. Cent. Nerv. Syst. Dis..

[52] Selkoe D.J. (2008). Biochemistry and molecular biology of amyloid β-protein and the mechanism of Alzheimer’s disease. Handb. Clin. Neurol..

[53] Yin J., Valin K.L., Dixon M.L., Leavenworth J.W. (2017). The role of microglia and macrophages in CNS homeostasis, autoimmunity, and cancer. J. Immunol. Res..

[54] Sarlus H., Heneka M.T. (2017). Microglia in Alzheimer’s disease. J. Clin. Investig..

[55] Hemonnot A.L., Hua J., Ulmann L., Hirbec H. (2019). Microglia in Alzheimer Disease: Well-known targets and new opportunities. Front. Aging Neurosci..

[56] Wyss-Coray T., Lin C., Yan F., Yu G.Q., Rohde M., McConlogue L., Masliah E., Mucke L. (2001). TGF-beta1 promotes microglial amyloid-beta clearance and reduces plaque burden in transgenic mice. Nat. Med..

[57] Mandrekar-Colucci S., Landreth G.E. (2010). Microglia and inflammation in Alzheimer’s disease. CNS Neurol. Disord. Drug Targets.

[58] Araque A., Navarrete M. (2010). Glial cells in neuronal network function. Philos. Trans. R. Soc. Lond. B Biol. Sci..

[59] Medeiros R., LaFerla F.M. (2013). Astrocytes: Conductors of the Alzheimer disease neuroinflammatory symphony. Exp. Neurol..

[60] Rodríguez-Arellano J.J., Parpura V., Zorec R., Verkhratsky A. (2016). Astrocytes in physiological aging and Alzheimer’s disease. Neuroscience.

[61] Kato S., Gondo T., Hoshii Y., Takahashi M., Yamada M., Ishihara T. (1998). Confocal observation of senile plaques in Alzheimer’s disease: Senile plaque morphology and relationship between senile plaques and astrocytes. Pathol. Int..

[62] Von Bernhardi R., Ramirez G. (2001). Microglia-astrocyte interaction in Alzheimer’s disease: Friends or foes for the nervous system?. Biol. Res..

[63] Nicoll J.A., Weller R.O. (2003). A new role for astrocytes: β-amyloid homeostasis and degradation. Trends Mol. Med..

[64] Liu C.C., Hu J., Zhao N., Wang J., Wang N., Cirrito J.R., Kanekiyo T., Holtzman D.M., Bu G. (2017). Astrocytic LRP1 mediates brain Aβ clearance and impacts amyloid deposition. J. Neurosci..

[65] Maragakis N.J., Rothstein J.D. (2006). Mechanisms of Disease: Astrocytes in neurodegenerative disease. Nat. Clin. Pract. Neurol..

[66] Chiarini A., Armato U., Liu D., Dal Prà I. (2017). Calcium-Sensing Receptor Antagonist NPS 2143 Restores Amyloid Precursor Protein Physiological Non-Amyloidogenic Processing in Aβ-Exposed Adult Human Astrocytes. Sci. Rep..

[67] Bushong E.A., Martone M.E., Jones Y.Z., Ellisman M.H. (2002). Protoplasmic astrocytes in CA1 stratum radiatum occupy separate anatomical domains. J. Neurosci..

[68] McIver S.R., Faideau M., Haydon P.G., Cui C., Grandison L., Noronha A. (2013). Astrocyte-neuron communications. Neural-Immune Interactions in Brain Function and Alcohol-Related Disorders.

[69] Antanitus D.S. (1998). A theory of cortical neuron-astrocyte interaction. Neuroscientist.

[70] Mawuenyega K.G., Sigurdson W., Ovod V., Munsell L., Kasten T., Morris J.C., Yarasheski K.E., Bateman R.J. (2010). Decreased clearance of CNS beta-amyloid in Alzheimer’s disease. Science.

[71] Kettenmann H., Ransom B.R. (2013). Neuroglia.

[72] González-Reyes R.E., Nava-Mesa M.O., Vargas-Sánchez K., Ariza-Salamanca D., Mora-Muñoz L. (2017). Involvement of astrocytes in Alzheimer’s Disease from a neuroinflammatory and oxidative stress perspective. Front. Mol. Neurosci..

[73] Kigerl K.A., Lai W., Rivest S., Hart R.P., Satoskar A.R., Popovich P.G. (2007). Toll-like receptor (TLR)-2 and TLR-4 regulate inflammation, gliosis, and myelin sparing after spinal cord injury. J. Neurochem..

[74] Lee G.S., Subramanian N., Kim A.I., Aksentijevich I., Goldbach-Mansky R., Sacks D.B., Germain R.N., Kastner D.L., Chae J.J. (2012). The calcium-sensing receptor regulates the NLRP3 inflammasome through Ca^2+^ and cAMP. Nature.

[75] Labbé K., Saleh M., Couillin I., Pétrilli V., Martinon F. (2011). Pyroptosis: A Caspase-1-dependent programmed cell death and a barrier to infection. The Inflammasomes. Progress in Inflammation Research.

[76] Lee M.S., Kim Y.J. (2007). Pattern-recognition receptor signaling initiated from extracellular, membrane, and cytoplasmic space. Mol. Cells..

[77] Thundyil J., Lim K.L. (2015). DAMPs and neurodegeneration. Ageing Res Rev..

[78] Schaefer L. (2014). Complexity of danger: The diverse nature of damage-associated molecular patterns. J. Biol. Chem..

[79] Santoni G., Cardinali C., Morelli M.B., Santoni M., Nabissi M., Amantini C. (2015). Danger- and pathogen-associated molecular patterns recognition by pattern-recognition receptors and ion channels of the transient receptor potential family triggers the inflammasome activation in immune cells and sensory neurons. J. Neuroinflammation.

[80] Jin H.S., Suh H.W., Kim S.J., Jo E.K. (2017). Mitochondrial control of innate immunity and inflammation. Immune Netw..

[81] Frevert C.W., Felgenhauer J., Wygrecka M., Nastase M.V., Schaefer L. (2018). Danger-associated molecular patterns derived from the extracellular matrix provide temporal control of innate immunity. J. Histochem. Cytochem..

[82] Gong T., Liu L., Jiang W., Zhou R. (2020). DAMP-sensing receptors in sterile inflammation and inflammatory diseases. Nat. Rev. Immunol..

[83] Verdier Y., Penke B. (2004). Binding sites of amyloid β-peptide in cell plasma membrane and implications for Alzheimer’s disease. Curr. Protein Pept. Sci..

[84] Prabhudas M., Bowdish D., Drickamer K., Febbraio M., Herz J., Kobzik L., Krieger M., Loike J., Means T.K., Moestrup S.K. (2014). Standardizing scavenger receptor nomenclature. J. Immunol..

[85] Colonna M. (2003). TREMs in the immune system and beyond. Nat. Rev. Immunol..

[86] Hickman S.E., El Khoury J. (2014). TREM2 and the neuroimmunology of Alzheimer’s disease. Biochem. Pharmacol..

[87] Klesney-Tait J., Turnbull I.R., Colonna M. (2006). The TREM receptor family and signal integration. Nat. Immunol..

[88] Cannon J.P., O’Driscoll M., Litman G.W. (2012). Specific lipid recognition is a general feature of CD300 and TREM molecules. Immunogenetics.

[89] Daws M.R., Sullam P.M., Niemi E.C., Chen T.T., Tchao N.K., Seaman W.E. (2003). Pattern recognition by TREM-2: Binding of anionic ligands. J. Immunol..

[90] Wang Y., Cella M., Mallinson K., Ulrich J.D., Young K.L., Robinette M.L., Gilfillan S., Krishnan G.M., Sudhakar S., Zinselmeyer B.H. (2015). TREM2 lipid sensing sustains the microglial response in an Alzheimer’s disease model. Cell.

[91] Takahashi K., Rochford C.D., Neumann H. (2005). Clearance of apoptotic neurons without inflammation by microglial triggering receptor expressed on myeloid cells-2. J. Exp. Med..

[92] Jiang T., Tan L., Zhu X.C., Zhang Q.Q., Cao L., Tan M.S., Gu L.Z., Wang H.F., Ding Z.Z., Zhang Y.D. (2014). Upregulation of TREM2 ameliorates neuropathology and rescues spatial cognitive impairment in a transgenic mouse model of Alzheimer’s disease. Neuropsychopharmacology.

[93] Kawabori M., Kacimi R., Kauppinen T., Calosing C., Kim J.Y., Hsieh C.L., Nakamura M.C., Yenari M.A. (2015). Triggering receptor expressed on myeloid cells 2 (TREM2) deficiency attenuates phagocytic activities of microglia and exacerbates ischemic damage in experimental stroke. J. Neurosci..

[94] Bisht K., Sharma K.P., Lecours C., Sánchez M.G., El Hajj H., Milior G., Olmos-Alonso A., Gómez-Nicola D., Luheshi G., Vallières L. (2016). Dark microglia: A new phenotype predominantly associated with pathological states. Glia.

[95] Mazaheri F., Snaidero N., Kleinberger G., Madore C., Daria A., Werner G., Krasemann S., Capell A., Trümbach D., Wurst W. (2017). TREM2 deficiency impairs chemotaxis and microglial responses to neuronal injury. EMBO Rep..

[96] Ulland T.K., Song W.M., Huang S.C., Ulrich J.D., Sergushichev A., Beatty W.L., Loboda A.A., Zhou Y., Cairns N.J., Kambal A. (2017). TREM2 maintains microglial metabolic fitness in Alzheimer’s disease. Cell.

[97] Poliani P.L., Wang Y., Fontana E., Robinette M.L., Yamanishi Y., Gilfillan S., Colonna M. (2015). TREM2 sustains microglial expansion during aging and response to demyelination. J. Clin. Investig..

[98] Yuan P., Condello C., Keene C.D., Wang Y., Bird T.D., Paul S.M., Luo W., Colonna M., Baddeley D., Grutzendler J. (2016). TREM2 haplodeficiency in mice and humans impairs the microglia barrier function leading to decreased amyloid compaction and severe axonal dystrophy. Neuron.

[99] Wang Y., Ulland T.K., Ulrich J.D., Song W., Tzaferis J.A., Hole J.T., Yuan P., Mahan T.E., Shi Y., Gilfillan S. (2016). TREM2-mediated early microglial response limits diffusion and toxicity of amyloid plaques. J. Exp. Med..

[100] Jay T.R., Hirsch A.M., Broihier M.L., Miller C.M., Neilson L.E., Ransohoff R.M., Lamb B.T., Landreth G.E. (2017). Disease progression-dependent effects of TREM2 deficiency in a mouse model of Alzheimer’s disease. J. Neurosci..

[101] Jiang T., Zhang Y.D., Chen Q., Gao Q., Zhu X.C., Zhou J.S., Shi J.Q., Lu H., Tan L., Yu J.T. (2016). TREM2 modifies microglial phenotype and provides neuroprotection in P301S tau transgenic mice. Neuropharmacology.

[102] Jiang T., Wan Y., Zhang Y.D., Zhou J.S., Gao Q., Zhu X.C., Shi J.Q., Lu H., Tan L., Yu J.T. (2017). TREM2 Overexpression has no improvement on neuropathology and cognitive impairment in aging APPswe/PS1dE9 mice. Mol. Neurobiol..

[103] Yeh F.L., Wang Y., Tom I., Gonzalez L.C., Sheng M. (2016). TREM2 binds to apolipoproteins, including APOE and CLU/APOJ, and thereby facilitates uptake of amyloid-beta by microglia. Neuron.

[104] Krasemann S., Madore C., Cialic R., Baufeld C., Calcagno N., El Fatimy R., Beckers L., O’Loughlin E., Xu Y., Fanek Z. (2017). The TREM2-APOE pathway drives the transcriptional phenotype of dysfunctional microglia in neurodegenerative diseases. Immunity.

[105] Keren-Shaul H., Spinrad A., Weiner A., Matcovitch-Natan O., Dvir-Szternfeld R., Ulland T.K., David E., Baruch K., Lara-Astaiso D., Toth B. (2017). A unique microglia type associated with restricting development of Alzheimer’s Disease. Cell.

[106] Wunderlich P., Glebov K., Kemmerling N., Tien N.T., Neumann H., Walter J. (2013). Sequential proteolytic processing of the triggering receptor expressed on myeloid cells-2 (TREM2) protein by ectodomain shedding and γ-secretase-dependent intramembranous cleavage. J. Biol. Chem..

[107] Schmid C.D., Sautkulis L.N., Danielson P.E., Cooper J., Hasel K.W., Hilbush B.S., Sutcliffe J.G., Carson M.J. (2002). Heterogeneous expression of the triggering receptor expressed on myeloid cells-2 on adult murine microglia. J. Neurochem..

[108] Piccio L., Deming Y., Del-Águila J.L., Ghezzi L., Holtzman D.M., Fagan A.M., Fenoglio C., Galimberti D., Borroni B., Cruchaga C. (2016). Cerebrospinal fluid soluble TREM2 is higher in Alzheimer disease and associated with mutation status. Acta Neuropathol..

[109] Heslegrave A., Heywood W., Paterson R., Magdalinou N., Svensson J., Johansson P., Öhrfelt A., Blennow K., Hardy J., Schott J. (2016). Increased cerebrospinal fluid soluble TREM2 concentration in Alzheimer’s disease. Mol. Neurodegener..

[110] Zhong L., Chen X.F., Wang T., Wang Z., Liao C., Wang Z., Huang R., Wang D., Li X., Wu L. (2017). Soluble TREM2 induces inflammatory responses and enhances microglial survival. J. Exp. Med..

[111] Bemiller S.M., McCray T.J., Allan K., Formica S.V., Xu G., Wilson G., Kokiko-Cochran O.N., Crish S.D., Lasagna-Reeves C.A., Ransohoff R.M. (2017). TREM2 deficiency exacerbates tau pathology through dysregulated kinase signaling in a mouse model of tauopathy. Mol. Neurodegener..

[112] Lill C.M., Rengmark A., Pihlstrom L., Fogh I., Shatunov A., Sleiman P.M., Wang L.S., Liu T., Lassen C.F., Meissner E. (2015). The role of TREM2 R47H as a risk factor for Alzheimer’s disease, frontotemporal lobar degeneration, amyotrophic lateral sclerosis, and Parkinson’s disease. Alzheimers Dement..

[113] Ayer A.H., Wojta K., Ramos E.M., Dokuru D., Chen J.A., Karydas A.M., Papatriantafyllou J.D., Agiomyrgiannakis D., Kamtsadeli V., Tsinia N. (2019). Frequency of the TREM2 R47H variant in various neurodegenerative disorders. Alzheimer Dis. Assoc. Disord..

[114] Ghezzi L., Carandini T., Arighi A., Fenoglio C., Arcaro M., De Riz M., Pietroboni A.M., Fumagalli G.G., Basilico P., Calvi A. (2017). Evidence of CNS β-amyloid deposition in Nasu-Hakola disease due to the *TREM2* Q33X mutation. Neurology.

[115] Borroni B., Ferrari F., Galimberti D., Nacmias B., Barone C., Bagnoli S., Fenoglio C., Piaceri I., Archetti S., Bonvicini C. (2014). Heterozygous TREM2 mutations in frontotemporal dementia. Neurobiol. Aging.

[116] Rayaprolu S., Mullen B., Baker M., Lynch T., Finger E., Seeley W.W., Hatanpaa K.J., Lomen-Hoerth C., Kertesz A., Bigio E.H. (2013). TREM2 in neurodegeneration: Evidence for association of the p.R47H variant with frontotemporal dementia and Parkinson’s disease. Mol. Neurodegener..

[117] Jiang T., Zhang Y.D., Gao Q., Zhou J.S., Zhu X.C., Lu H., Shi J.Q., Tan L., Chen Q., Yu J.T. (2016). TREM1 facilitates microglial phagocytosis of amyloid beta. Acta Neuropathol..

[118] Maillard-Lefebvre H., Boulanger E., Daroux M., Gaxatte C., Hudson B.I., Lambert M. (2009). Soluble receptor for advanced glycation end products: A new biomarker in diagnosis and prognosis of chronic inflammatory diseases. Rheumatology.

[119] Huttunen H.J., Kuja-Panula J., Sorci G., Agneletti A.L., Donato R., Rauvala H. (2000). Coregulation of neurite outgrowth and cell survival by amphoterin and S100 proteins through receptor for advanced glycation end products (RAGE) activation. J. Biol. Chem..

[120] Chuah Y.K., Basir R., Talib H., Tie T.H., Nordin N. (2013). Receptor for advanced glycation end products and its involvement in inflammatory diseases. Int. J Inflam..

[121] MacLean M., Derk J., Ruiz H.H., Juranek J.K., Ramasamy R., Schmidt A.M. (2019). The Receptor for Advanced Glycation End Products (RAGE) and DIAPH1: Implications for vascular and neuroinflammatory dysfunction in disorders of the central nervous system. Neurochem. Int..

[122] Ibrahim Z.A., Armour C.L., Phipps S., Sukkar M.B. (2013). RAGE and TLRs: Relatives, friends or neighbours?. Mol. Immunol..

[123] Shekhtman A., Ramasamy R., Schmidt A.M. (2017). Glycation & the RAGE axis: Targeting signal transduction through DIAPH1. Expert Rev. Proteom..

[124] Chen G.Y., Nuñez G. (2010). Sterile inflammation: Sensing and reacting to damage. Nat. Rev. Immunol..

[125] Vénéreau E., Ceriotti C., Bianchi M.E. (2015). DAMPs from cell death to new life. Front. Immunol..

[126] Land W.G. (2015). The role of Damage-Associated Molecular Patterns in human diseases: Part I—Promoting inflammation and immunity. Sultan Qaboos Univ. Med. J..

[127] Festoff B.W., Sajja R.K., van Dreden P., Cucullo L. (2016). HMGB1 and thrombin mediate the blood-brain barrier dysfunction acting as biomarkers of neuroinflammation and progression to neurodegeneration in Alzheimer’s disease. J. Neuroinflammation.

[128] Xie J., Méndez J.D., Méndez-Valenzuela V., Aguilar-Hernández M.M. (2013). Cellular signalling of the receptor for advanced glycation end products (RAGE). Cell Signal..

[129] Gasparotto J., Girardi C.S., Somensi N., Ribeiro C.T., Moreira J., Michels M., Sonai B., Rocha M., Steckert A.V., Barichello T. (2018). Receptor for advanced glycation end products mediates sepsis-triggered amyloid-β accumulation, Tau phosphorylation, and cognitive impairment. J. Biol. Chem..

[130] Fournet M., Bonté F., Desmoulière A. (2018). Glycation damage: A possible hub for major pathophysiological disorders and aging. Aging Dis..

[131] Chung M.M., Nicol C.J., Cheng Y.C., Lin K.H., Chen Y.L., Pei D., Lin C.H., Shih Y.N., Yen C.H., Chen S.J. (2017). Metformin activation of AMPK suppresses AGE-induced inflammatory response in hNSCs. Exp. Cell Res..

[132] Sawikr Y., Yarla N.S., Peluso I., Kamal M.A., Aliev G., Bishayee A. (2017). Neuroinflammation in Alzheimer’s Disease: The preventive and therapeutic potential of polyphenolic nutraceuticals. Adv. Protein Chem. Struct. Biol..

[133] Bierhaus A., Humpert P.M., Morcos M., Wendt T., Chavakis T., Arnold B., Stern D.M., Nawroth P.P. (2005). Understanding RAGE, the receptor for advanced glycation end products. J. Mol. Med..

[134] Schmidt A.M., Yan S.D., Yan S.F., Stern D.M. (2000). The biology of the receptor for advanced glycation end products and its ligands. Biochim. Biophys. Acta.

[135] Barnes P.J., Karin M. (1997). Nuclear factor-kappaB: A pivotal transcription factor in chronic inflammatory diseases. N. Engl. J. Med..

[136] Fann D.Y., Lim Y.A., Cheng Y.L., Lok K.Z., Chunduri P., Baik S.H., Drummond G.R., Dheen S.T., Sobey C.G., Jo D.G. (2018). Evidence that NF-κB and MAPK signaling promotes NLRP inflammasome activation in neurons following ischemic stroke. Mol. Neurobiol..

[137] Yao D., Brownlee M. (2010). Hyperglycemia-induced reactive oxygen species increase expression of the receptor for advanced glycation end products (RAGE) and RAGE ligands. Diabetes.

[138] Gonzalez-Reyes R.E., Rubiano M.G. (2018). Astrocyte’s RAGE: More than just a question of mood. Cent. Nerv. Syst. Agents Med. Chem..

[139] Ponath G., Schettler C., Kaestner F., Voigt B., Wentker D., Arolt V., Rothermundt M. (2007). Autocrine S100B effects on astrocytes are mediated via RAGE. J. Neuroimmunol..

[140] Wang Z., Li D.D., Liang Y.Y., Wang D.S., Cai N.S. (2002). Activation of astrocytes by advanced glycation end products: Cytokines induction and nitric oxide release. Acta Pharmacol. Sin..

[141] Sparvero L.J., Asafu-Adjei D., Kang R., Tang D., Amin N., Im J., Rutledge R., Lin B., Amoscato A.A., Zeh H.J. (2009). RAGE (Receptor for Advanced Glycation Endproducts), RAGE ligands, and their role in cancer and inflammation. J. Transl. Med..

[142] Villarreal A., Seoane R., González Torres A., Rosciszewski G., Angelo M.F., Rossi A., Barker P.A., Ramos A.J. (2014). S100B protein activates a RAGE-dependent autocrine loop in astrocytes: Implications for its role in the propagation of reactive gliosis. J. Neurochem..

[143] Kaur D., Sharma V., Deshmukh R. (2019). Activation of microglia and astrocytes: A roadway to neuroinflammation and Alzheimer’s disease. Inflammopharmacology.

[144] Nonaka S., Nakanishi H. (2019). Microglial clearance of focal apoptotic synapses. Neurosci. Lett..

[145] Sasaki N., Toki S., Chowei H., Saito T., Nakano N., Hayashi Y., Takeuchi M., Makita Z. (2001). Immunohistochemical distribution of the receptor for advanced glycation end products in neurons and astrocytes in Alzheimer’s disease. Brain Res..

[146] Criscuolo C., Fontebasso V., Middei S., Stazi M., Ammassari-Teule M., Yan S.S., Origlia N. (2017). Entorhinal Cortex dysfunction can be rescued by inhibition of microglial RAGE in an Alzheimer’s disease mouse model. Sci. Rep..

[147] Prasad K. (2019). AGE-RAGE stress: A changing landscape in pathology and treatment of Alzheimer’s disease. Mol. Cell. Biochem..

[148] Choi B.R., Cho W.H., Kim J., Lee H.J., Chung C., Jeon W.K., Han J.S. (2014). Increased expression of the receptor for advanced glycation end products in neurons and astrocytes in a triple transgenic mouse model of Alzheimer’s disease. Exp. Mol. Med..

[149] Fang F., Yu Q., Arancio O., Chen D., Gore S.S., Yan S.S., Yan S.F. (2018). RAGE mediates Aβ accumulation in a mouse model of Alzheimer’s disease via modulation of β- and γ-secretase activity. Hum. Mol. Genet..

[150] Batkulwar K., Godbole R., Banarjee R., Kassaar O., Williams R.J., Kulkarni M.J. (2018). Advanced Glycation End products modulate amyloidogenic APP processing and Tau phosphorylation: A mechanistic link between glycation and the development of Alzheimer’s Disease. ACS Chem. Neurosci..

[151] Du H., Li P., Wang J., Qing X., Li W. (2012). The interaction of amyloid β and the receptor for advanced glycation endproducts induces matrix metalloproteinase-2 expression in brain endothelial cells. Cell Mol. Neurobiol..

[152] Yan S.D., Bierhaus A., Nawroth P.P., Stern D.M. (2009). RAGE and Alzheimer’s disease: A progression factor for amyloid-β-induced cellular perturbation?. J. Alzheimers Dis..

[153] Askarova S., Yang X., Sheng W., Sun G.Y., Lee J.C. (2011). Role of Aβ-receptor for advanced glycation endproducts interaction in oxidative stress and cytosolic phospholipase A₂ activation in astrocytes and cerebral endothelial cells. Neuroscience.

[154] Emi M., Asaoka H., Matsumoto A., Itakura H., Kurihara Y., Wada Y., Kanamori H., Yazaki Y., Takahashi E., Lepert M. (1993). Structure, organization, and chromosomal mapping of the human macrophage scavenger receptor gene. J. Biol. Chem..

[155] Kelley J.L., Ozment T.R., Li C., Schweitzer J.B., Williams D.L. (2014). Scavenger receptor-A (CD204): A two-edged sword in health and disease. Crit. Rev. Immunol..

[156] Gough P.J., Greaves D.R., Gordon S. (1998). A naturally occurring isoform of the human macrophage scavenger receptor (SR-A) gene generated by alternative splicing blocks modified LDL uptake. J. Lipid Res..

[157] Yu B., Cheng C., Wu Y., Guo L., Kong D., Zhang Z., Wang Y., Zheng E., Liu Y., He Y. (2020). Interactions of ferritin with scavenger receptor class A members. J. Biol. Chem..

[158] El Khoury J.B., Moore K.J., Means T.K., Leung J., Terada K., Toft M., Freeman M.W., Luster A.D. (2003). CD36 mediates the innate host response to beta-amyloid. J. Exp. Med..

[159] Zhang H., Su Y.J., Zhou W.W., Wang S.W., Xu P.X., Yu X.L., Liu R.T. (2014). Activated scavenger receptor A promotes glial internalization of aβ. PLoS ONE.

[160] Hickman S.E., Allison E.K., El Khoury J. (2008). Microglial dysfunction and defective beta-amyloid clearance pathways in aging Alzheimer’s disease mice. J. Neurosci..

[161] Christie R.H., Freeman M., Hyman B.T. (1996). Expression of the macrophage scavenger receptor, a multifunctional lipoprotein receptor, in microglia associated with senile plaques in Alzheimer’s disease. Am. J Pathol..

[162] Yu Y., Ye R.D. (2015). Microglial Aβ receptors in Alzheimer’s disease. Cell. Mol. Neurobiol..

[163] Plüddemann A., Neyen C., Gordon S. (2007). Macrophage scavenger receptors and host-derived ligands. Methods.

[164] Mukhopadhyay S., Varin A., Chen Y., Liu B., Tryggvason K., Gordon S. (2011). SR-A/MARCO-mediated ligand delivery enhances intracellular TLR and NLR function, but ligand scavenging from cell surface limits TLR4 response to pathogens. Blood.

[165] Novakowski K.E., Huynh A., Han S., Dorrington M.G., Yin C., Tu Z., Pelka P., Whyte P., Guarné A., Sakamoto K. (2016). A naturally occurring transcript variant of MARCO reveals the SRCR domain is critical for function. Immunol. Cell Biol..

[166] Xu J., Flaczyk A., Neal L.M., Fa Z., Eastman A.J., Malachowski A.N., Cheng D., Moore B.B., Curtis J.L., Osterholzer J.J. (2017). Scavenger Receptor MARCO Orchestrates Early Defenses and Contributes to Fungal Containment during Cryptococcal Infection. J. Immunol..

[167] Pittman R.C., Knecht T.P., Rosenbaum M.S., Taylor C.A. (1987). A non endocytotic mechanism for the selective uptake of high density lipoprotein-associated cholesterol esters. J. Biol. Chem..

[168] Shen W.J., Asthana S., Kraemer F.B., Azhar S. (2018). Scavenger receptor B type 1: Expression, molecular regulation, and cholesterol transport function. J. Lipid Res..

[169] Shen W.J., Azhar S., Kraemer F.B. (2018). SR-B1: A unique multifunctional receptor for cholesterol influx and efflux. Annu. Rev. Physiol..

[170] Reina S.A., Llabre M.M., Allison M.A., Wilkins J.T., Mendez A.J., Arnan M.K., Schneiderman N., Sacco R.L., Carnethon M., Delaney J.A.C. (2015). HDL cholesterol and stroke risk: The multi-ethnic study of atherosclerosis. Atherosclerosis.

[171] Linton M.F., Tao H., Linton E.F., Yancey P.G. (2017). SR-BI: A Multifunctional Receptor in Cholesterol Homeostasis and Atherosclerosis. Trends Endocrinol. Metab..

[172] Ohgami N., Miyazaki A., Sakai M., Kuniyasu A., Nakayama H., Horiuchi S. (2003). Advanced glycation end products (AGE) inhibit scavenger receptor class B type I-mediated reverse cholesterol transport: A new crossroad of AGE to cholesterol metabolism. J. Atheroscler. Thromb..

[173] Lescoat A., Ballerie A., Lelong M., Augagneur Y., Morzadec C., Jouneau S., Jégo P., Fardel O., Vernhet L., Lecureur V. (2020). Crystalline silica impairs efferocytosis abilities of human and mouse macrophages: Implication for silica-associated systemic sclerosis. Front. Immunol..

[174] Sekhar V., Pollicino T., Diaz G., Engle R.E., Alayli F., Melis M., Kabat J., Tice A., Pomerenke A., Altan-Bonnet N. (2018). Infection with hepatitis C virus depends on TACSTD2, a regulator of claudin-1 and occludin highly downregulated in hepatocellular carcinoma. PLoS Pathog..

[175] Iram T., Trudler D., Kain D., Kanner S., Galron R., Vassar R., Barzilai A., Blinder P., Fishelson Z., Frenkel D. (2016). Astrocytes from old Alzheimer’s disease mice are impaired in Aβ uptake and in neuroprotection. Neurobiol. Dis..

[176] Thanopoulou K., Fragkouli A., Stylianopoulou F., Georgopoulos S. (2010). Scavenger receptor class B type I (SR-BI) regulates perivascular macrophages and modifies amyloid pathology in an Alzheimer mouse model. Proc. Natl. Acad. Sci. USA.

[177] Febbraio M., Hajjar D.P., Silverstein R.L. (2001). CD36: A class B scavenger receptor involved in angiogenesis, atherosclerosis, inflammation and lipid metabolism. J. Clin. Investig..

[178] Leitinger N. (2003). Oxidized phospholipids as modulators of inflammation in atherosclerosis. Curr. Opin. Lipidol..

[179] Coraci I.S., Husemann J., Berman J.W., Hulette C., Dufour J.H., Campanella G.K., Luster A.D., Silverstein S.C., El-Khoury J.B. (2002). CD36, a class B scavenger receptor, is expressed on microglia in Alzheimer’s disease brains and can mediate production of reactive oxygen species in response to beta-amyloid fibrils. Am. J. Pathol..

[180] Sheedy F.J., Grebe A., Rayner K.J., Kalantari P., Ramkhelawon B., Carpenter S.B., Becker C.E., Ediriweera H.N., Mullick A.E., Golenbock D.T. (2013). CD36 coordinates NLRP3 inflammasome activation by facilitating intracellular nucleation of soluble ligands into particulate ligands in sterile inflammation. Nat. Immunol..

[181] Bamberger M.E., Harris M.E., McDonald D.R., Husemann J., Landreth G.E. (2003). A cell surface receptor complex for fibrillar beta-amyloid mediates microglial activation. J. Neurosci..

[182] Husemann J., Silverstein S.C. (2001). Expression of scavenger receptor class B, type I, by astrocytes and vascular smooth muscle cells in normal adult mouse and human brain and in Alzheimer’s disease brain. Am. J. Pathol..

[183] Husemann J., Loike J.D., Anankov R., Febbraio M., Silverstein S.C. (2002). Scavenger receptors in neurobiology and neuropathology: Their role on microglia and other cells of the nervous system. Glia.

[184] Alarcón R., Fuenzalida C., Santibáñez M., von Bernhardi R. (2005). Expression of scavenger receptors in glial cells. Comparing the adhesion of astrocytes and microglia from neonatal rats to surface-bound β-amyloid. J. Biol. Chem..

[185] Mulder S.D., Veerhuis R., Blankenstein M.A., Nielsen H.M. (2012). The effect of amyloid associated proteins on the expression of genes involved in amyloid-β clearance by adult human astrocytes. Exp. Neurol..

[186] Thal D.R. (2012). The role of astrocytes in amyloid β-protein toxicity and clearance. Exp. Neurol..

[187] Eugenín J., Vecchiola A., Murgas P., Arroyo P., Cornejo F., von Bernhardi R. (2016). Expression pattern of scavenger receptors and amyloid-β phagocytosis of astrocytes and microglia in culture are modified by acidosis: Implications for Alzheimer’s Disease. J. Alzheimers Dis..

[188] Barton G.M., Medzhitov R. (2003). Toll-like receptor signaling pathways. Science.

[189] Crack P.J., Bray P.J. (2007). Toll-like receptors in the brain and their potential roles in neuropathology. Immunol. Cell Biol..

[190] Hopkins P.A., Sriskandan S. (2005). Mammalian Toll-like receptors: To immunity and beyond. Clin. Exp. Immunol..

[191] Jana M., Palencia C.A., Pahan K. (2008). Fibrillar amyloid-beta peptides activate microglia via TLR2: Implications for Alzheimer’s disease. J. Immunol..

[192] Letiembre M., Liu Y., Walter S., Hao W., Pfander T., Wrede A., Schulz-Schaeffer W., Fassbender K. (2009). Screening of innate immune receptors in neurodegenerative diseases: A similar pattern. Neurobiol. Aging.

[193] Liu Y., Walter S., Stagi M., Cherny D., Letiembre M., Schulz-Schaeffer W., Heine H., Penke B., Neumann H., Fassbender K. (2005). LPS receptor (CD14): A receptor for phagocytosis of Alzheimer’s amyloid peptide. Brain.

[194] Walter S., Letiembre M., Liu Y., Heine H., Penke B., Hao W., Bode B., Manietta N., Walter J., Schulz-Schuffer W. (2007). Role of the toll-like receptor 4 in neuroinflammation in Alzheimer’s disease. Cell. Physiol. Biochem..

[195] Liu S., Liu Y., Hao W., Wolf L., Kiliaan A.J., Penke B., Rübe C.E., Walter J., Heneka M.T., Hartmann T. (2012). TLR2 is a primary receptor for Alzheimer’s amyloid β peptide to trigger neuroinflammatory activation. J. Immunol..

[196] Balducci C., Frasca A., Zotti M., La Vitola P., Mhillaj E., Grigoli E., Iacobellis M., Grandi F., Messa M., Colombo L. (2017). Toll-like receptor 4-dependent glial cell activation mediates the impairment in memory establishment induced by β-amyloid oligomers in an acute mouse model of Alzheimer’s disease. Brain Behav. Immun..

[197] Tahara K., Kim H.D., Jin J.J., Maxwell J.A., Li L., Fukuchi K. (2006). Role of toll-like receptor signalling in Abeta uptake and clearance. Brain.

[198] Tauber S.C., Ebert S., Weishaupt J.H., Reich A., Nau R., Gerber J. (2009). Stimulation of Toll-like receptor 9 by chronic intraventricular unmethylated cytosine-guanine DNA infusion causes neuroinflammation and impaired spatial memory. J. Neuropathol. Exp. Neurol..

[199] Kufer T.A., Sansonetti P.J. (2007). Sensing of bacteria: NOD a lonely job. Curr. Opin. Microbiol..

[200] Ye Z., Ting J.P. (2008). NLR, the nucleotide-binding domain leucine-rich repeat containing gene family. Curr. Opin. Immunol..

[201] Boivin A., Pineau I., Barrette B., Filali M., Vallières N., Rivest S., Lacroix S. (2007). Toll-like receptor signaling is critical for Wallerian degeneration and functional recovery after peripheral nerve injury. J. Neurosci..

[202] Kigerl K.A., de Rivero Vaccari J.P., Dietrich W.D., Popovich P.G., Keane R.W. (2014). Pattern recognition receptors and central nervous system repair. Exp. Neurol..

[203] De Rivero Vaccari J.P., Minkiewicz J., Wang X., de Rivero Vaccari J.C., German R., Marcillo A.E., Dietrich W.D., Keane R.W. (2012). Astrogliosis involves activation of retinoic acid-inducible gene-like signaling in the innate immune response after spinal cord injury. Glia.

[204] Kufer T.A., Sansonetti P.J. (2011). NLR functions beyond pathogen recognition. Nat. Immunol..

[205] Huber R.G., Eibl C., Fuchs J.E. (2015). Intrinsic flexibility of NLRP pyrin domains is a key factor in their conformational dynamics, fold stability, and dimerization. Protein Sci..

[206] Kufer T.A., Kremmer E., Banks D.J., Philpott D.J. (2006). Role for Erbin in Bacterial Activation of Nod2. Infect. Immun..

[207] Schroder K., Tschopp J. (2010). The inflammasomes. Cell.

[208] Gross O., Thomas C.J., Guarda G., Tschopp J. (2011). The inflammasome: An integrated view. Immunol. Rev..

[209] Davis B.K., Wen H., Ting J.P. (2011). The inflammasome NLRs in immunity, inflammation, and associated diseases. Annu. Rev. Immunol..

[210] Minkiewicz J., de Rivero Vaccari J.P., Keane R.W. (2013). Human astrocytes express a novel NLRP2 inflammasome. Glia.

[211] Vladimer G.I., Weng D., Paquette S.W., Vanaja S.K., Rathinam V.A., Aune M.H., Conlon J.E., Burbage J.J., Proulx M.K., Liu Q. (2012). The NLRP12 inflammasome recognizes Yersinia pestis. Immunity.

[212] Walsh J.G., Muruve D.A., Power C. (2014). Inflammasomes in the CNS. Nat. Rev. Neurosci..

[213] Vande Walle L., Lamkanfi M. (2016). Pyroptosis. Curr. Biol..

[214] McKenzie B.A., Mamik M.K., Saito L.B., Boghozian R., Monaco M.C., Major E.O., Lu J.Q., Branton W.G., Power C. (2018). Caspase-1 inhibition prevents glial inflammasome activation and pyroptosis in models of multiple sclerosis. Proc. Natl. Acad. Sci. USA.

[215] Kaushal V., Dye R., Pakavathkumar P., Foveau B., Flores J., Hyman B., Ghetti B., Koller B.H., LeBlanc A.C. (2015). Neuronal NLRP1 inflammasome activation of Caspase-1 coordinately regulates inflammatory interleukin-1-beta production and axonal degeneration-associated Caspase-6 activation. Cell Death Differ..

[216] Freeman L., Guo H., David C.N., Brickey W.J., Jha S., Ting J.P. (2017). NLR members NLRC4 and NLRP3 mediate sterile inflammasome activation in microglia and astrocytes. J. Exp. Med..

[217] Yap J.K.Y., Pickard B.S., Chan E.W.L., Gan S.Y. (2019). The role of neuronal NLRP1 inflammasome in Alzheimer’s disease: Bringing neurons into the neuroinflammation game. Mol. Neurobiol..

[218] Nyúl-Tóth Á., Kozma M., Nagyőszi P., Nagy K., Fazakas C., Haskó J., Molnár K., Farkas A.E., Végh A.G., Váró G. (2017). Expression of pattern recognition receptors and activation of the non-canonical inflammasome pathway in brain pericytes. Brain Behav. Immun..

[219] Nagyőszi P., Nyúl-Tóth Á., Fazakas C., Wilhelm I., Kozma M., Molnár J., Haskó J., Krizbai I.A. (2015). Regulation of NOD-like receptors and inflammasome activation in cerebral endothelial cells. J. Neurochem..

[220] Abulafia D.P., de Rivero Vaccari J.P., Lozano J.D., Lotocki G., Keane R.W., Dietrich W.D. (2009). Inhibition of the inflammasome complex reduces the inflammatory response after thromboembolic stroke in mice. J. Cereb. Blood Flow Metab..

[221] De Rivero Vaccari J.P., Lotocki G., Marcillo A.E., Dietrich W.D., Keane R.W. (2008). A molecular platform in neurons regulates inflammation after spinal cord injury. J. Neurosci..

[222] Lechan R.M., Toni R., Clark B.D., Cannon J.G., Shaw A.R., Dinarello C.A., Reichlin S. (1990). Immunoreactive interleukin-1 beta localization in the rat forebrain. Brain Res..

[223] Heneka M.T. (2017). Inflammasome activation and innate immunity in Alzheimer’s disease. Brain Pathol..

[224] Martinon F., Burns K., Tschopp J. (2002). The inflammasome: A molecular platform triggering activation of inflammatory caspases and processing of proIL-beta. Mol. Cell.

[225] Finger J.N., Lich J.D., Dare L.C., Cook M.N., Brown K.K., Duraiswami C., Bertin J., Gough P.J. (2012). Autolytic proteolysis within the function to find domain (FIIND) is required for NLRP1 inflammasome activity. J. Biol. Chem..

[226] Franchi L., Eigenbrod T., Muñoz-Planillo R., Nuñez G. (2009). The inflammasome: A caspase-1-activation platform that regulates immune responses and disease pathogenesis. Nat. Immunol..

[227] White C.S., Lawrence C.B., Brough D., Rivers-Auty J. (2017). Inflammasomes as therapeutic targets for Alzheimer’s disease. Brain Pathol..

[228] Fann D.Y., Lee S.Y., Manzanero S., Tang S.C., Gelderblom M., Chunduri P., Bernreuther C., Glatzel M., Cheng Y.L., Thundyil J. (2013). Intravenous immunoglobulin suppresses NLRP1 and NLRP3 inflammasome-mediated neuronal death in ischemic stroke. Cell Death Dis..

[229] Tan M.S., Tan L., Jiang T., Zhu X.C., Wang H.F., Jia C.D., Yu J.T. (2014). Amyloid-β induces NLRP1-dependent neuronal pyroptosis in models of Alzheimer’s disease. Cell Death Dis..

[230] Saadi M., Karkhah A., Pourabdolhossein F., Ataie A., Monif M., Nouri H.R. (2020). Involvement of NLRC4 inflammasome through caspase-1 and IL-1β augments neuroinflammation and contributes to memory impairment in an experimental model of Alzheimer’s like disease. Brain Res. Bull..

[231] Healy L.M., Yaqubi M., Ludwin S., Antel J.P. (2020). Species differences in immune-mediated CNS tissue injury and repair: A (neuro)inflammatory topic. Glia.

[232] Kinoshita T., Wang Y., Hasegawa M., Imamura R., Suda T. (2005). PYPAF3, a PYRIN-containing APAF-1-like protein, is a feedback regulator of caspase-1-dependent interleukin-1beta secretion. J. Biol. Chem..

[233] Vizlin-Hodzic D., Zhai Q., Illes S., Södersten K., Truvé K., Parris T.Z., Sobhan P.K., Salmela S., Kosalai S.T., Kanduri C. (2017). Early onset of inflammation during ontogeny of bipolar disorder: The NLRP2 inflammasome gene distinctly differentiates between patients and healthy controls in the transition between iPS cell and neural stem cell stages. Transl. Psychiatry.

[234] Peng H., Chang B., Lu C., Su J., Wu Y., Lv P., Wang Y., Liu J., Zhang B., Quan F. (2012). Nlrp2, a maternal effect gene required for early embryonic development in the mouse. PLoS ONE.

[235] Sun X., Song X., Zhang L., Sun J., Wei X., Meng L., An J. (2016). NLRP2 is highly expressed in a mouse model of ischemic stroke. Biochem. Biophys. Res. Commun..

[236] Zhang Q., Sun Y., He Z., Xu Y., Li X., Ding J., Lu M., Hu G. (2020). Kynurenine regulates NLRP2 inflammasome in astrocytes and its implications in depression. Brain Behav. Immun..

[237] Bruey J.M., Bruey-Sedano N., Newman R., Chandler S., Stehlik C., Reed J.C. (2004). PAN1/NALP2/PYPAF2, an inducible inflammatory mediator that regulates NF-kappaB and caspase-1 activation in macrophages. J. Biol. Chem..

[238] Mangan M.S.J., Olhava E.J., Roush W.R., Seidel H.M., Glick G.D., Latz E. (2018). Targeting the NLRP3 inflammasome in inflammatory diseases. Nat. Rev. Drug Discov..

[239] Leemans J.C., Cassel S.L., Sutterwala F.S. (2011). Sensing damage by the NLRP3 inflammasome. Immunol. Rev..

[240] Zhou R., Yazdi A.S., Menu P., Tschopp J. (2011). A role for mitochondria in NLRP3 inflammasome activation. Nature.

[241] Iyer S.S., He Q., Janczy J.R., Elliott E.I., Zhong Z., Olivier A.K., Sadler J.J., Knepper-Adrian V., Han R., Qiao L. (2013). Mitochondrial cardiolipin is required for Nlrp3 inflammasome activation. Immunity.

[242] Zhong Z., Liang S., Sanchez-Lopez E., He F., Shalapour S., Lin X.J., Wong J., Ding S., Seki E., Schnabl B. (2018). New mitochondrial DNA synthesis enables NLRP3 inflammasome activation. Nature.

[243] Katsnelson M.A., Rucker L.G., Russo H.M., Dubyak G.R. (2015). K+ efflux agonists induce NLRP3 inflammasome activation independently of Ca^2+^ signaling. J. Immunol..

[244] Gong T., Jiang W., Zhou R. (2018). Control of inflammasome activation by phosphorylation. Trends Biochem. Sci..

[245] Choi A.J., Ryter S.W. (2014). Inflammasomes: Molecular regulation and implications for metabolic and cognitive diseases. Mol. Cells.

[246] Elliott E.I., Sutterwala F.S. (2015). Initiation and perpetuation of NLRP3 inflammasome activation and assembly. Immunol. Rev..

[247] Murakami T., Ockinger J., Yu J., Byles V., McColl A., Hofer A.M., Horng T. (2012). Critical role for calcium mobilization in activation of the NLRP3 inflammasome. Proc. Natl. Acad. Sci. USA.

[248] Rossol M., Pierer M., Raulien N., Quandt D., Meusch U., Rothe K., Schubert K., Schöneberg T., Schaefer M., Krügel U. (2012). Extracellular Ca2+ is a danger signal activating the NLRP3 inflammasome through G protein-coupled calcium sensing receptors. Nat. Commun..

[249] Chen J., Chen Z.J. (2018). PtdIns4P on dispersed trans-Golgi network mediates NLRP3 inflammasome activation. Nature.

[250] Sokolowska M., Chen L.Y., Liu Y., Martinez-Anton A., Qi H.Y., Logun C., Alsaaty S., Park Y.H., Kastner D.L., Chae J.J. (2015). Prostaglandin E2 Inhibits NLRP3 Inflammasome Activation through EP4 Receptor and Intracellular Cyclic AMP in Human Macrophages. J. Immunol..

[251] Halle A., Hornung V., Petzold G.C., Stewart C.R., Monks B.G., Reinheckel T., Fitzgerald K.A., Latz E., Moore K.J., Golenbock D.T. (2008). The NALP3 inflammasome is involved in the innate immune response to amyloid-beta. Nat. Immunol..

[252] Heneka M.T., Kummer M.P., Stutz A., Delekate A., Schwartz S., Vieira-Saecker A., Griep A., Axt D., Remus A., Tzeng T.C. (2013). NLRP3 is activated in Alzheimer’s disease and contributes to pathology in APP/PS1 mice. Nature.

[253] Thangavel R., Stolmeier D., Yang X., Anantharam P., Zaheer A. (2012). Expression of glia maturation factor in neuropathological lesions of Alzheimer’s disease. Neuropathol. Appl. Neurobiol..

[254] Thangavel R., Bhagavan S.M., Ramaswamy S.B., Surpur S., Govindarajan R., Kempuraj D., Zaheer S., Raikwar S., Ahmed M.E., Selvakumar G.P. (2018). Co-Expression of Glia Maturation factor and Apolipoprotein E4 in Alzheimer’s Disease Brain. J. Alzheimers Dis..

[255] Ahmed M.E., Iyer S., Thangavel R., Kempuraj D., Selvakumar G.P., Raikwar S.P., Zaheer S., Zaheer A. (2017). Co-Localization of Glia Maturation Factor with NLRP3 Inflammasome and Autophagosome Markers in Human Alzheimer’s Disease Brain. J. Alzheimers Dis..

[256] Ramaswamy S.B., Bhagavan S.M., Kaur H., Giler G.E., Kempuraj D., Thangavel R., Ahmed M.E., Selvakumar G.P., Raikwar S.P., Zaheer S. (2019). Glia Maturation Factor in the Pathogenesis of Alzheimer’s disease. Open Access J. Neurol. Neurosurg..

[257] Couturier J., Stancu I.C., Schakman O., Pierrot N., Huaux F., Kienlen-Campard P., Dewachter I., Octave J.N. (2016). Activation of phagocytic activity in astrocytes by reduced expression of the inflammasome component ASC and its implication in a mouse model of Alzheimer disease. J. Neuroinflammation.

[258] Saresella M., La Rosa F., Piancone F., Zoppis M., Marventano I., Calabrese E., Rainone V., Nemni R., Mancuso R., Clerici M. (2016). The NLRP3 and NLRP1 inflammasomes are activated in Alzheimer’s disease. Mol. Neurodegener..

[259] Feng J., Wang J.X., Du Y.H., Liu Y., Zhang W., Chen J.F., Liu Y.J., Zheng M., Wang K.J., He G.Q. (2018). Dihydromyricetin inhibits microglial activation and neuroinflammation by suppressing NLRP3 inflammasome activation in APP/PS1 transgenic mice. CNS Neurosci. Ther..

[260] Dempsey C., Rubio Araiz A., Bryson K.J., Finucane O., Larkin C., Mills E.L., Robertson A., Cooper M.A., O’Neill L., Lynch M.A. (2017). Inhibiting the NLRP3 inflammasome with MCC950 promotes non-phlogistic clearance of amyloid-β and cognitive function in APP/PS1 mice. Brain Behav. Immun..

[261] Ebrahimi T., Rust M., Kaiser S.N., Slowik A., Beyer C., Koczulla A.R., Schulz J.B., Habib P., Bach J.P. (2018). α1-antitrypsin mitigates NLRP3-inflammasome activation in amyloid β1-42-stimulated murine astrocytes. J. Neuroinflammation.

[262] Stancu I.C., Cremers N., Vanrusselt H., Couturier J., Vanoosthuyse A., Kessels S., Lodder C., Brône B., Huaux F., Octave J.N. (2019). Aggregated Tau activates NLRP3-ASC inflammasome exacerbating exogenously seeded and non-exogenously seeded Tau pathology in vivo. Acta Neuropathol..

[263] Ising C., Venegas C., Zhang S., Scheiblich H., Schmidt S.V., Vieira-Saecker A., Schwartz S., Albasset S., McManus R.M., Tejera D. (2019). NLRP3 inflammasome activation drives tau pathology. Nature.

[264] Murphy N., Grehan B., Lynch M.A. (2014). Glial uptake of amyloid beta induces NLRP3 inflammasome formation via cathepsin-dependent degradation of NLRP10. Neuromolecular Med..

[265] Lu B., Nakamura T., Inouye K., Li J., Tang Y., Lundbäck P., Valdes-Ferrer S.I., Olofsson P.S., Kalb T., Roth J. (2012). Novel role of PKR in inflammasome activation and HMGB1 release. Nature.

[266] He Y., Franchi L., Núñez G. (2013). The protein kinase PKR is critical for LPS-induced iNOS production but dispensable for inflammasome activation in macrophages. Eur. J. Immunol..

[267] Rathinam V.A., Jiang Z., Waggoner S.N., Sharma S., Cole L.E., Waggoner L., Vanaja S.K., Monks B.G., Ganesan S., Latz E. (2010). The AIM2 inflammasome is essential for host defense against cytosolic bacteria and DNA viruses. Nat. Immunol..

[268] Matyszewski M., Morrone S.R., Sohn J. (2018). Digital signaling network drives the assembly of the AIM2-ASC inflammasome. Proc. Natl. Acad. Sci. USA.

[269] Wu P.J., Hung Y.F., Liu H.Y., Hsueh Y.P. (2017). Deletion of the inflammasome sensor Aim2 mitigates Aβ deposition and microglial activation but increases inflammatory cytokine expression in an Alzheimer Disease mouse model. Neuroimmunomodulation.

[270] Zhao Y., Yang J., Shi J., Gong Y.N., Lu Q., Xu H., Liu L., Shao F. (2011). The NLRC4 inflammasome receptors for bacterial flagellin and type III secretion apparatus. Nature.

[271] Bauer R., Rauch I. (2020). The NAIP/NLRC4 inflammasome in infection and pathology. Mol. Aspects Med..

[272] Mariathasan S., Newton K., Monack D.M., Vucic D., French D.M., Lee W., Roose-Girma M., Erickson S., Dixit V.M. (2004). Differential activation of the inflammasome by caspase-1 adaptors ASC and Ipaf. Nature.

[273] Qu Y., Misaghi S., Izrael-Tomasevic A., Newton K., Gilmour L.L., Lamkanfi M., Louie S., Kayagaki N., Liu J., Kömüves L. (2012). Phosphorylation of NLRC4 is critical for inflammasome activation. Nature.

[274] Christie L.A., Su J.H., Tu C.H., Dick M.C., Zhou J., Cotman C.W. (2007). Differential regulation of inhibitors of apoptosis proteins in Alzheimer’s disease brains. Neurobiol. Dis..

[275] Lesné S., Gabriel C., Nelson D.A., White E., Mackenzie E.T., Vivien D., Buisson A. (2005). Akt-dependent expression of NAIP-1 protects neurons against amyloid-{beta} toxicity. J. Biol. Chem..

[276] Seidl R., Bajo M., Böhm K., LaCasse E.C., MacKenzie A.E., Cairns N., Lubec G. (1999). Neuronal apoptosis inhibitory protein (NAIP)-like immunoreactivity in brains of adult patients with Down syndrome. J. Neural Transm. Suppl..

[277] Liu L., Chan C. (2014). IPAF inflammasome is involved in interleukin-1β production from astrocytes, induced by palmitate; implications for Alzheimer’s Disease. Neurobiol. Aging..

[278] Marwarha G., Schommer J., Lund J., Schommer T., Ghribi O. (2018). Palmitate-induced C/EBP homologous protein activation leads to NF-κB-mediated increase in BACE1 activity and amyloid beta genesis. J. Neurochem..

[279] Chen K., Zhang L., Huang J., Gong W., Dunlop N.M., Wang J.M. (2008). Cooperation between NOD2 and Toll-like receptor 2 ligands in the up-regulation of mouse mFPR2, a G-protein-coupled Abeta42 peptide receptor, in microglial cells. J. Leukoc. Biol..

[280] Fani Maleki A., Cisbani G., Plante M.M., Préfontaine P., Laflamme N., Gosselin J., Rivest S. (2020). Muramyl dipeptide-mediated immunomodulation on monocyte subsets exerts therapeutic effects in a mouse model of Alzheimer’s disease. J. Neuroinflammation.

[281] Li Z.G., Shui S.F., Han X.W., Yan L. (2020). NLRP10 ablation protects against ischemia/reperfusion-associated brain injury by suppression of neuroinflammation. Exp. Cell Res..

[282] Zeng Q., Hu C., Qi R., Lu D. (2018). PYNOD reduces microglial inflammation and consequent neurotoxicity upon lipopolysaccharides stimulation. Exp. Ther. Med..

[283] Shichita T., Hasegawa E., Kimura A., Morita R., Sakaguchi R., Takada I., Sekiya T., Ooboshi H., Kitazono T., Yanagawa T. (2012). Peroxiredoxin family proteins are key initiators of post-ischemic inflammation in the brain. Nat. Med..

[284] Gringhuis S.I., Kaptein T.M., Wevers B.A., Theelen B., van der Vlist M., Boekhout T., Geijtenbeek T.B. (2012). Dectin-1 is an extracellular pathogen sensor for the induction and processing of IL-1β via a noncanonical caspase-8 inflammasome. Nat. Immunol..

[285] Gensel J.C., Wang Y., Guan Z., Beckwith K.A., Braun K.J., Wei P., McTigue D.M., Popovich P.G. (2015). Toll-Like Receptors and Dectin-1, a C-Type Lectin Receptor, Trigger Divergent Functions in CNS Macrophages. J. Neurosci..

[286] Ye X.C., Hao Q., Ma W.J., Zhao Q.C., Wang W.W., Yin H.H., Zhang T., Wang M., Zan K., Yang X.X. (2020). Dectin-1/Syk signaling triggers neuroinflammation after ischemic stroke in mice. J. Neuroinflammation.

[287] Baldwin K.T., Carbajal K.S., Segal B.M., Giger R.J. (2015). Neuroinflammation triggered by β-glucan/dectin-1 signaling enables CNS axon regeneration. Proc. Natl. Acad. Sci. USA.

[288] Webster J.A., Gibbs J.R., Clarke J., Ray M., Zhang W., Holmans P., Rohrer K., Zhao A., Marlowe L., Kaleem M. (2009). Genetic control of human brain transcript expression in Alzheimer disease. Am. J. Hum. Genet..

[289] Friedman B.A., Srinivasan K., Ayalon G., Meilandt W.J., Lin H., Huntley M.A., Cao Y., Lee S.H., Haddick P., Ngu H. (2018). Diverse brain myeloid expression profiles reveal distinct microglial activation states and aspects of Alzheimer’s Disease not evident in mouse models. Cell Rep..

[290] Wes P.D., Easton A., Corradi J., Barten D.M., Devidze N., DeCarr L.B., Truong A., He A., Barrezueta N.X., Polson C. (2014). Tau overexpression impacts a neuroinflammation gene expression network perturbed in Alzheimer’s disease. PLoS ONE.

[291] Holtman I.R., Raj D.D., Miller J.A., Schaafsma W., Yin Z., Brouwer N., Wes P.D., Möller T., Orre M., Kamphuis W. (2015). Induction of a common microglia gene expression signature by aging and neurodegenerative conditions: A co-expression meta-analysis. Acta Neuropathol. Commun..

[292] Wang X., Liu G.J., Gao Q., Li N., Wang R.T. (2020). C-type lectin-like receptor 2 and zonulin are associated with mild cognitive impairment and Alzheimer’s disease. Acta Neurol. Scand..

[293] Patel J.R., García-Sastre A. (2014). Activation and regulation of pathogen sensor RIG-I. Cytokine Growth Factor Rev..

[294] De Rivero Vaccari J.P., Brand F.J., Sedaghat C., Mash D.C., Dietrich W.D., Keane R.W. (2014). RIG-1 receptor expression in the pathology of Alzheimer’s disease. J. Neuroinflammation.

[295] Chazal M., Beauclair G., Gracias S., Najburg V., Simon-Lorière E., Tangy F., Komarova A.V., Jouvenet N. (2018). RIG-I recognizes the 5’ Region of Dengue and Zika virus genomes. Cell Rep..

[296] Johnson M.B., Halman J.R., Burmeister A.R., Currin S., Khisamutdinov E.F., Afonin K.A., Marriott I. (2020). Retinoic acid inducible gene-I mediated detection of bacterial nucleic acids in human microglial cells. J. Neuroinflammation.

[297] Zheng B., Wang X., Liu Y., Li Y., Long S., Gu C., Ye J., Xie S., Cao S. (2019). Japanese Encephalitis Virus infection induces inflammation of swine testis through RIG-I-NF-ĸB signaling pathway. Vet. Microbiol..

[298] Li L., Yang R., Feng M., Guo Y., Wang Y., Guo J., Lu X. (2018). Rig-I is involved in inflammation through the IPS-1/TRAF6 pathway in astrocytes under chemical hypoxia. Neurosci. Lett..

[299] Chiarini A., Armato U., Dal Prà I. (2019). Family C G-Protein-Coupled Receptors in Alzheimer’s Disease and Therapeutic Implications. Front. Pharmacol..

[300] Brown E.M. (2002). The pathophysiology of primary hyperparathyroidism. J. Bone Miner. Res..

[301] Breitwieser G.E. (2013). The calcium sensing receptor life cycle: Trafficking, cell surface expression, and degradation. Best Pract. Res. Clin. Endocrinol. Metab..

[302] Chiarini A., Armato U., Gardenal E., Gui L., Dal Prà I. (2017). Amyloid β-Exposed Human Astrocytes Overproduce Phospho-Tau and Overrelease It within Exosomes, Effects Suppressed by Calcilytic NPS 2143-Further Implications for Alzheimer’s Therapy. Front. Neurosci..

[303] Leach K., Gregory K.J., Kufareva I., Khajehali E., Cook A.E., Abagyan R., Conigrave A.D., Sexton P.M., Christopoulos A. (2016). Towards a structural understanding of allosteric drugs at the human calcium-sensing receptor. Cell Res..

[304] Nemeth E.F., Goodman W.G. (2016). Calcimimetic and calcilytic drugs: Feats, flops, and futures. Calcif. Tissue Int..

[305] Chakravarty B., Chattopadhyay N., Brown E.M. (2012). Signaling through the extracellular calcium-sensing receptor (CaSR). Adv. Exp. Med. Biol..

[306] Zhang C., Miller C.L., Brown E.M., Yang J.J. (2015). The calcium sensing receptor: From calcium sensing to signaling. Sci. China Life Sci..

[307] Hofer A.M., Brown E.M. (2003). Extracellular calcium sensing and signalling. Nat. Rev. Mol. Cell Biol..

[308] Klein G.L., Castro S.M., Garofalo R.P. (2016). The calcium-sensing receptor as a mediator of inflammation. Semin. Cell Dev. Biol..

[309] Yarova P.L., Stewart A.L., Sathish V., Britt R.D., Thompson M.A.P., Lowe A.P., Freeman M., Aravamudan B., Kita H., Brennan S.C. (2015). Calcium-sensing receptor antagonists abrogate airway hyperresponsiveness and inflammation in allergic asthma. Sci. Transl. Med..

[310] Zhang X., Hong S., Qi S., Liu W., Zhang X., Shi Z., Chen W., Zhao M., Yin X. (2019). NLRP3 Inflammasome is involved in Calcium-Sensing Receptor-induced aortic remodeling in SHRs. Mediat. Inflamm..

[311] Lee J.W., Park H.A., Kwon O.K., Park J.W., Lee G., Lee H.J., Lee S.J., Oh S.R., Ahn K.S. (2017). NPS 2143, a selective calcium-sensing receptor antagonist inhibits lipopolysaccharide-induced pulmonary inflammation. Mol. Immunol..

[312] Jäger E., Murthy S., Schmidt C., Hahn M., Strobel S., Peters A., Stäubert C., Sungur P., Venus T., Geisler M. (2020). Calcium-sensing receptor-mediated NLRP3 inflammasome response to calciprotein particles drives inflammation in rheumatoid arthritis. Nat. Commun..

[313] Mattar P., Bravo-Sagua R., Tobar N., Fuentes C., Troncoso R., Breitwieser G., Lavandero S., Cifuentes M. (2018). Autophagy mediates calcium-sensing receptor-induced TNFα production in human preadipocytes. Biochim. Biophys. Acta Mol. Basis Dis..

[314] Hu B., Tong F., Xu L., Shen Z., Yan L., Xu G., Shen R. (2018). Role of Calcium Sensing Receptor in streptozotocin-induced diabetic rats exposed to renal ischemia reperfusion injury. Kidney Blood Press. Res..

[315] Iamartino L., Elajnaf T., Kallay E., Schepelmann M. (2018). Calcium-sensing receptor in colorectal inflammation and cancer: Current insights and future perspectives. World J. Gastroenterol..

[316] Bernichtein S., Pigat N., Barry Delongchamps N., Boutillon F., Verkarre V., Camparo P., Reyes-Gomez E., Méjean A., Oudard S.M., Lepicard E.M. (2017). Vitamin D3 prevents calcium-induced progression of early-stage prostate tumors by counteracting TRPC6 and Calcium Sensing Receptor upregulation. Cancer Res..

[317] Olszak I.T., Poznansky M.C., Evans R.H., Olson D., Kos C., Pollak M.R., Brown E.M., Scadden D.T. (2000). Extracellular calcium elicits a chemokinetic response from monocytes in vitro and in vivo. J. Clin. Investig..

[318] Hendy G.N., Canaff L. (2016). Calcium-Sensing Receptor Gene: Regulation of Expression. Front. Physiol..

[319] Bagur R., Hajnoczky G. (2017). Intracellular Ca(2+) sensing: Its role in calcium homeostasis and signaling. Mol. Cell.

[320] Hendy G.N., Canaff L. (2016). Calcium-sensing receptor, proinflammatory cytokines and calcium homeostasis. Semin. Cell. Dev. Biol..

[321] Canton J., Schlam D., Breuer C., Gütschow M., Glogauer M., Grinstein S. (2016). Calcium-sensing receptors signal constitutive macropinocytosis and facilitate the uptake of NOD2 ligands in macrophages. Nat. Commun..

[322] Bandyopadhyay S., Tfelt-Hansen J., Chattopadhyay N. (2010). Diverse roles of extracellular calcium-sensing receptor in the central nervous system. J. Neurosci. Res..

[323] Yano S., Brown E.M., Chattopadhyay N. (2004). Calcium-sensing receptor in the brain. Cell Calcium.

[324] Chattopadhyay N., Ye C., Yamaguchi T., Nakai M., Kifor O., Vassilev P.M., Nishimura R.N., Brown E.M. (1999). The extracellular calcium-sensing receptor is expressed in rat microglia and modulates an outward K+ channel. J. Neurochem..

[325] Riccardi D., Kemp P.J. (2012). The calcium-sensing receptor beyond extracellular calcium homeostasis: Conception, development, adult physiology, and disease. Annu. Rev. Physyiol..

[326] Ruat M., Traiffort E. (2013). Roles of the calcium sensing receptor in the central nervous system. Best Pract. Res. Clin. Endocrinol. Metab..

[327] Noh J.S., Pak H.J., Shin Y.J., Riew T.R., Park J.H., Moon Y.W., Lee M.Y. (2015). Differential expression of the calcium-sensing receptor in the ischemic and border zones after transient focal cerebral ischemia in rats. J. Chem. Neuroanat..

[328] Guo Y., Yang X., He J., Liu J., Yang S., Dong H. (2018). Important roles of the Ca2+-sensing receptor in vascular health and disease. Life Sci..

[329] Conigrave A.D., Hampson D.R. (2006). Broad-spectrum L-amino acid sensing by class 3 G-protein-coupled receptors. Trends Endocrinol. Metab..

[330] Zhen Y., Ding C., Sun J., Wang Y., Li S., Dong L. (2016). Activation of the calcium-sensing receptor promotes apoptosis by modulating the JNK/p38 MAPK pathway in focal cerebral ischemia-reperfusion in mice. Am. J. Transl. Res..

[331] Wang C., Jia Q., Sun C., Jing C. (2020). Calcium sensing receptor contribute to early brain injury through the CaMKII/NLRP3 pathway after subarachnoid hemorrhage in mice. Biochem. Biophys. Res. Commun..

[332] Kim J.Y., Kim N., Yenari M.A., Chang W. (2013). Hypothermia and pharmacological regimens that prevent overexpression and overactivity of the extracellular calcium-sensing receptor protect neurons against traumatic brain injury. J. Neurotrauma.

[333] Bai S., Mao M., Tian L., Yu Y., Zeng J., Ouyang K., Yu L., Li L., Wang D., Deng X. (2015). Calcium sensing receptor mediated the excessive generation of β-amyloid peptide induced by hypoxia in vivo and in vitro. Biochem. Biophys. Res. Commun..

[334] Gardenal E., Chiarini A., Armato U., Dal Prà I., Verkhratsky A., Rodríguez J.J. (2017). Increased Calcium-Sensing Receptor Immunoreactivity in the Hippocampus of a Triple Transgenic Mouse Model of Alzheimer’s Disease. Front. Neurosci..

[335] Feng C., Bao X., Shan L., Ling Y., Ding Y., Wang J., Cao Y., Wang Q., Cui W., Xu S. (2020). Calcium-Sensing Receptor Mediates β-amyloid-induced synaptic formation impairment and cognitive deficits via regulation of cytosolic Phospholipase A2/Prostaglandin E2 metabolic pathway. Front. Aging Neurosci..

[336] Dal Prà I., Chiarini A., Nemeth E.F., Armato U., Whitfield J.F. (2005). Roles of Ca^2+^ and the Ca^2+^-sensing receptor (CaSR) in the expression of inducible NOS (nitric oxide synthase)-2 and its BH4 (tetrahydrobiopterin)-dependent activation in cytokine-stimulated adult human astrocytes. J. Cell. Biochem..

[337] Li Y.N., Pan R., Qin X.J., Yang W.L., Qi Z., Liu W., Liu K.J. (2014). Ischemic neurons activate astrocytes to disrupt endothelial barrier via increasing VEGF expression. J. Neurochem..

[338] Sabbagh M.N., Agro A., Bell J., Aisen P.S., Schweizer E., Galasko D. (2011). PF-04494700, an oral inhibitor of receptor for advanced glycation end products (RAGE), in Alzheimer disease. Alzheimer Dis. Assoc. Disord..

[339] Wang S., Mustafa M., Yuede C.M., Salazar S.V., Kong P., Long H., Ward M., Siddiqui O., Paul R., Gilfillan S. (2020). Anti-human TREM2 induces microglia proliferation and reduces pathology in an Alzheimer’s disease model. J. Exp. Med..

[340] Coll R.C., Robertson A.A., Chae J.J., Higgins S.C., Muñoz-Planillo R., Inserra M.C., Vetter I., Dungan L.S., Monks B.G., Stutz A. (2015). A small-molecule inhibitor of the NLRP3 inflammasome for the treatment of inflammatory diseases. Nat. Med..

[341] Yamanaka M., Ishikawa T., Griep A., Axt D., Kummer M.P., Heneka M.T. (2012). PPARγ/RXRα-induced and CD36-mediated microglial amyloid-β phagocytosis results in cognitive improvement in amyloid precursor protein/presenilin 1 mice. J. Neurosci..

[342] Mattson M.P. (2010). ER calcium and Alzheimer’s disease: In a state of flux. Sci. Signal..

